# CITK modulates BRCA1 recruitment at DNA double strand breaks sites through HDAC6

**DOI:** 10.1038/s41419-025-07655-4

**Published:** 2025-04-20

**Authors:** Giorgia Iegiani, Gianmarco Pallavicini, Alex Pezzotta, Alessia Brix, Alessia Ferraro, Marta Gai, Enrica Boda, Stephanie L. Bielas, Anna Pistocchi, Ferdinando Di Cunto

**Affiliations:** 1 Neuroscience Institute Cavalieri Ottolenghi, Turin, Italy; 2 Department of Neuroscience ‘Rita Levi Montalcini’, University of Turin, Torino, Italy; 3 Department of Medical Biotechnology and Translational Medicine, University of Milan, Milano, Italy; 4 Department of Molecular Biotechnology and Health Sciences, University of Turin, Torino, Italy; 5 Department of Human Genetics, University of Michigan Medical School, Ann Arbor, MI USA; 6 Neuroscience Graduate Program, University of Michigan Medical School, Ann Arbor, MI USA; 7 Department of Pediatrics, University of Michigan Medical School, Ann Arbor, MI USA

**Keywords:** Cell biology, Molecular biology

## Abstract

Citron Kinase (CITK) is a protein encoded by the *CIT* gene, whose pathogenic variants underlie microcephalic phenotypes that characterize MCPH17 syndrome. In neural progenitors, CITK loss leads to microtubule instability, resulting in mitotic spindle positioning defects, cytokinesis failure, and accumulation of DNA double strand breaks (DSBs), ultimately resulting in TP53-dependent senescence and apoptosis. Although DNA damage accumulation has been associated with impaired homologous recombination (HR), the role of CITK in this process and whether microtubule dynamics are involved is still unknown. In this report we show that CITK is required for proper BRCA1 localization at sites of DNA DSBs. We found that CITK’s scaffolding, rather than its catalytic activity, is necessary for maintaining BRCA1 interphase levels in progenitor cells during neurodevelopment. CITK regulates the nuclear levels of HDAC6, a modulator of both microtubule stability and DNA damage repair. Targeting HDAC6 in CITK-deficient cells increases microtubule stability and recovers BRCA1 localization defects and DNA damage levels to that detected in controls. In addition, the CIT-HDAC6 axis is functionally relevant in a MCPH17 zebrafish model, as HDAC6 targeting recovers the head size phenotype produced by interfering with the *CIT* orthologue gene. These data provide novel insights into the functional interplay between HR and microtubule dynamics and into the pathogenesis of CITK based MCPH17, which may be relevant for development of therapeutic strategies.

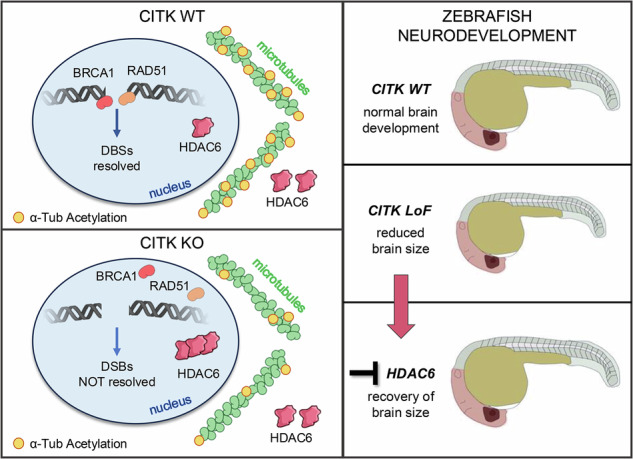

## Introduction

Brain development is dependent on rapid and persistent proliferation of progenitor cells that generate the high variety of neuronal subtypes. Maintenance of genomic integrity is critical for this process, since replication-associated DNA strand breaks occur with high frequency during proliferation of progenitor cells and neurogenesis [1]. A buildup of DNA double strand breaks (DSBs) is toxic for progenitor cells, as they can lead to chromosome rearrangements and mutations resulting in cell death [2]. Repair of DSBs may occurs by either homologous recombination (HR) or non-homologous end joining (NHEJ) [1]. Genomic fidelity during replication is strongly dependent on HR, that is ongoing from mid S through G2 phase. This process is relatively slow and error-free as sister chromatids serve as a template for repair [3]. HR is initiated when DSBs are resolved into single-stranded DNA (ssDNA) molecules by the MRN complex. This permits recessed ends to be bound by RPA complexes, which are removed and replaced by RAD51 via a BRCA1/BRCA2-dependent process. RAD51-bound ssDNA aligns with homologous sequences on the sister chromatid, to generate new DNA and repair the DSB lesion [3–5].

The developing brain is highly sensitive to DNA damage, as is evident by the pathogenic effect of defective DNA repair machinery in severe neurological disorders [6], which highlights the need to maintain genomic integrity during development. This is particularly evident in microcephaly [1, 7], a condition characterized by reduced head circumference of at least two standard deviations below the mean [8]. Primary hereditary microcephaly (MCPH) is a genetic form of microcephaly mostly caused by recessive mutations in single genes, 30 of which have been so far identified [9]. Accumulation of DNA damage is found in many MCPH models and several MCPH genes encode molecular machinery required for DNA repair and genomic stability [9, 10]. One of these genes is *Citron Kinase* (*CIT*), a recessive etiology of MCPH17 syndrome, which encodes the Citron Kinase protein (CITK) [11–14]. CITK loss leads to DNA damage accumulation and chromosomal aberrations in the developing brain of both mammals and Drosophila [15, 16]. Moreover, CITK loss increases the sensitivity of cells to ionizing radiation [17] and is important for HR-dependent DNA repair, in particular for the recruitment of RAD51 at DSBs lesions [15, 17, 18]. In addition, CITK is required during mitosis for correct cytokinesis completion, midbody positioning and abscission [15, 19–22]. Regulation of the latter processes is strongly dependent on the capability of CITK to promote microtubule stability [22, 23].

Microtubules play central roles not only during cell division, where they are the crucial components of the mitotic spindle [24, 25] but also for genome maintenance during interphase [26–28]. Several works have shown that microtubule stabilization is required for efficient DNA repair, revealing a link between microtubule dynamics and the DNA damage response [29–32]. In this work we addressed more in depth the mechanisms by which CITK affects HR, as well as their relationships with microtubule dynamics.

## Results

### CITK knockdown impairs BRCA1 recruitment at DSBs

**Fig. 1:**
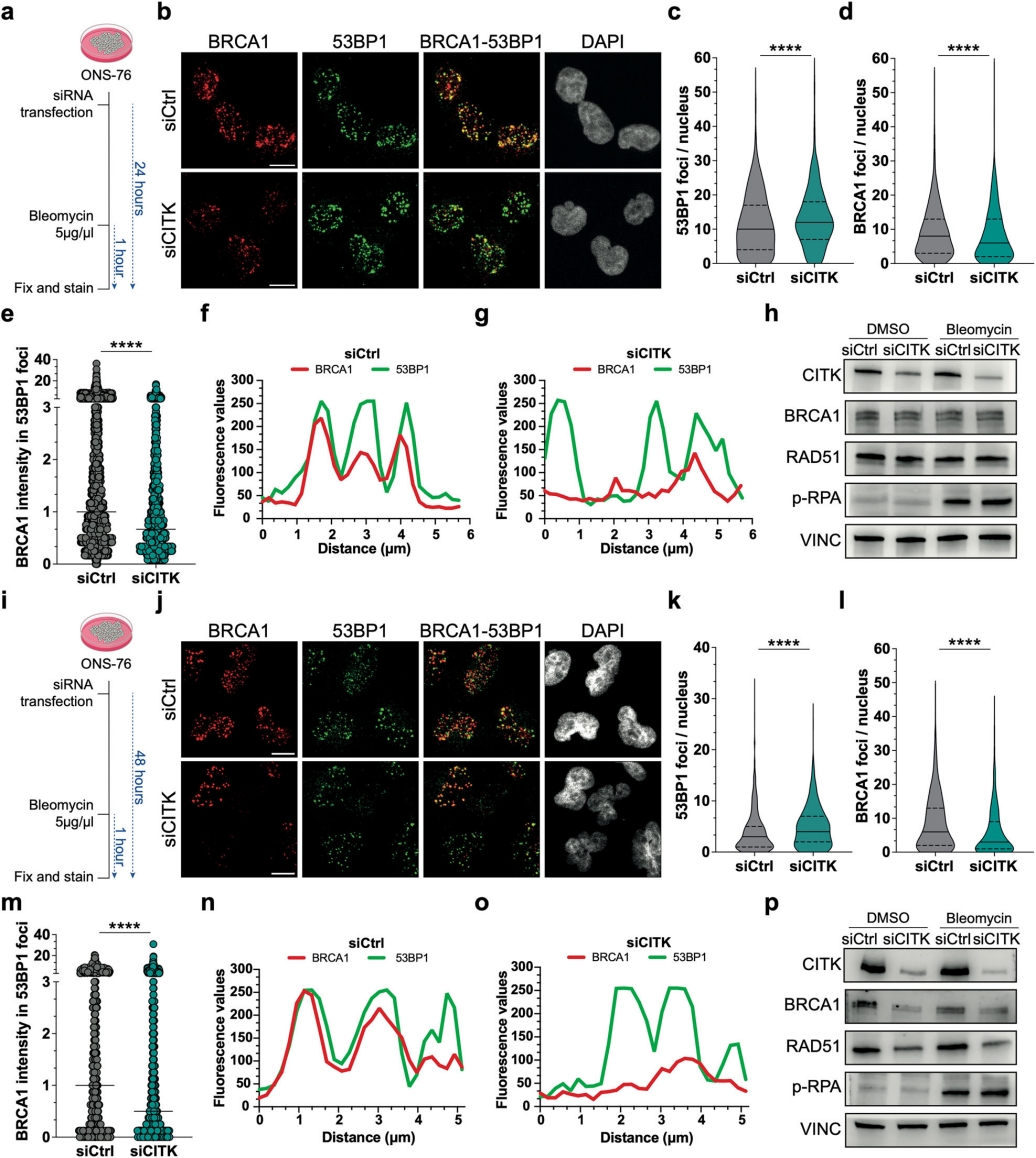
CITK knockdown reduces BRCA1 levels and colocalization between BRCA1 and 53BP1 after induction of DNA damage. **a**, **i** Schematic representation of the experiments performed. **b**, **j** Representative confocal images of ONS-76 cells treated as highlighted in (**a***,* **i**), respectively, immunostained for BRCA1 and 53BP1 and counterstained with DAPI. Scale bars: 10 μm. **c**, **d**, **k**, **l** Quantification of 53BP1 and BRCA1 foci per nucleus, in cells treated as in (**b**, **j**), respectively. **e**, **m** Quantification of BRCA1 signal intensity in each 53BP1 focus, in cells treated as in (**b**, **j**), respectively, after normalization to the median of control samples. **f**, **g**, **n**, **o** Examples of co-localization profiles between BRCA1 and 53BP1 signals in ONS-76 cells treated as (**b**, **j**), respectively. Fluorescence intensity was plotted for the two channels along a 6-μm-long line, randomly drawn in the nuclei of exemplar cells. **h**, **p** Western blot analysis of total lysate from ONS-76, obtained at the end of experiments performed as highlighted in (**a**, **i**), respectively. The levels of CITK, BRCA1, RAD51, phosphor-RPA were analyzed, and the internal loading control was Vinculin (VINC). All quantifications were based on at least four independent biological replicates; >300 cells were analyzed per condition in each replicate. ****P < 0.0001; Mann– Whitney U test.

During HR, RAD51 replaces RPA complex bound to ssDNA through a BRCA1-BRCA2 dependent processes [3–5]. CITK loss impairs HR and RAD51 recruitment at DSBs sites, without altering phospho-RPA levels [15, 17, 18], suggesting CITK loss may affect BRCA1 function too. To address this question, we took advantage of ONS-76 medulloblastoma cells, previously shown to exhibit a CITK-dependent sensitivity to DNA damage repair [17, 18]. We downregulated CITK expression by RNAi [17, 18] and induced DSBs accumulation with Bleomycin (Fig. 1a). As early as 24 h post-transfection, relative to controls, CITK-depleted cells accumulated 53BP1-labled nuclear foci (Fig. 1b, c), a reliable marker of DSBs increase [33]. This contrasted with a reduced number of BRCA1 foci per nucleus, (Fig. 1b–d). The intensity of BRCA1 signal co-localizing with 53BP1 foci was also significantly reduced (Fig. 1e–g). In line with previous publications [18], at this time point the expression of CITK was already downregulated and phospho-RPA (pRPA) was induced by Bleomycin treatment (Fig. 1h). In contrast, total levels of BRCA1 and RAD51 proteins, as well as their mRNA levels, were not significantly affected (Fig. 1h and Supplementary Fig. 1a). 48 h after siRNAs transfection, the effects were even more prominent than at 24 h, with increased number of 53BP1-positive foci, the reduction of BRCA1-positive foci and the reduced colocalization of BRCA1 signal with 53BP1 foci (Fig. 1i–o). However, at this time point, also the levels of BRCA1 and RAD51 proteins and transcripts were significantly down-regulated (Fig. 1p and Supplementary Fig. 1b). In line with the reduced protein levels, at 48 h after siRNA transfection we observed a reduction in BRCA1 colocalization with γ-H2AX foci, a marker of single and double strand DNA breaks [34, 35] (Supplementary Fig. 1f–h). This reduction was not observed at 24 h (Supplementary Fig. 1c–e). Taken together, these results indicate that CITK is required for the efficient recruitment of BRCA1 at DSBs provoked by DNA damaging agents.

**Fig. 2:**
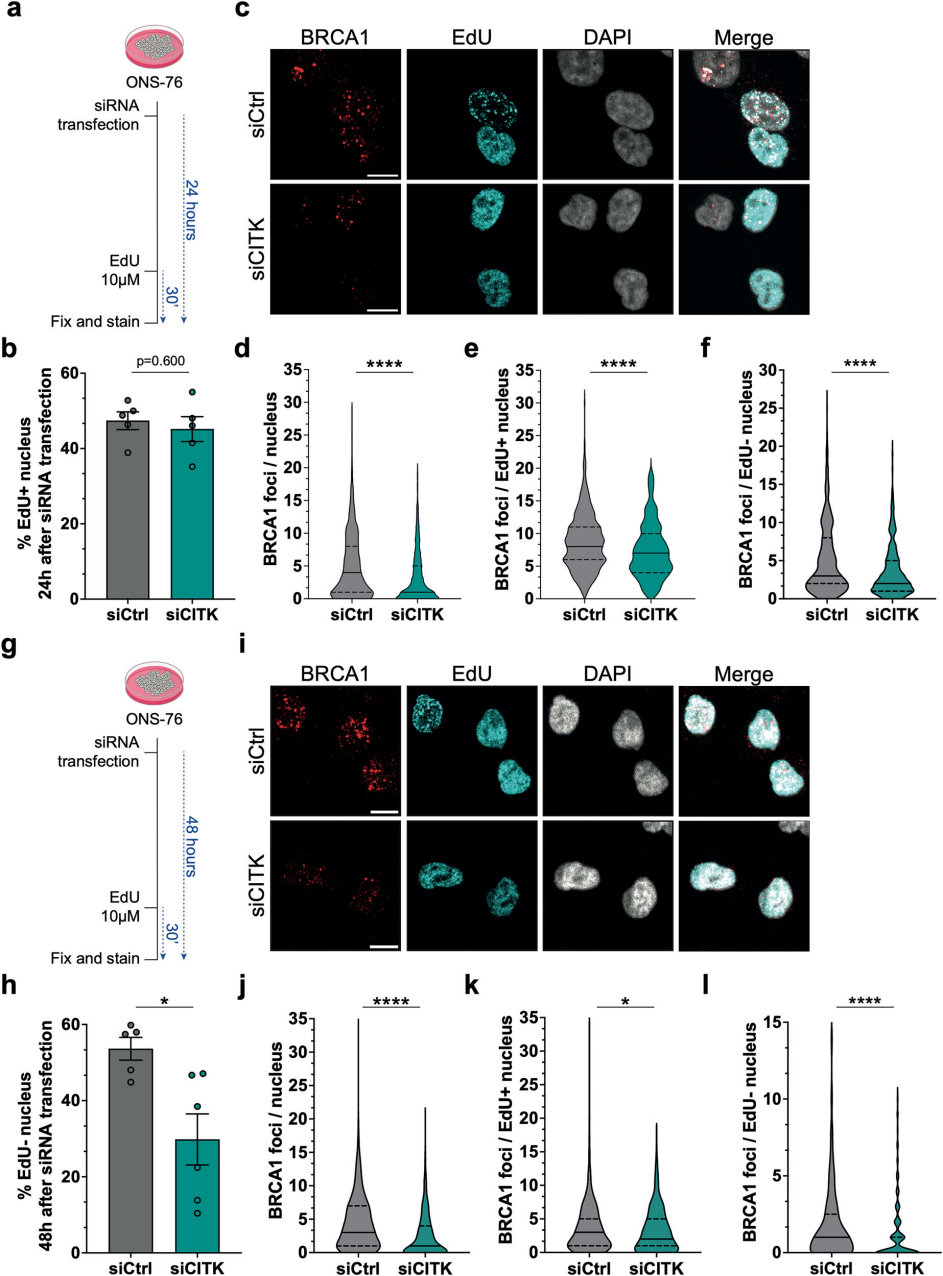
CIT-K knockdown reduces BRCA1 levels independently from proliferative state. **a**, **g** Schematic representation of the experiments performed. Quantification of the percentage of EdU positive ONS-76 cells 24 h (**b**) or 48 h (**h**) after transfection of the indicated siRNAs. Representative images of ONS-76 cells analyzed 24 h (**c**) or 48 h (**i**) after siRNAs transfection and treatment with 10 μM EdU for 30’, to label cells in S-phase. Cells were immunostained for BRCA1 and nuclei were counterstained with DAPI. Scale bars: 10 μm. **d**, **j** Quantification of BRCA1 foci per nucleus, in samples treated as in (**c**, **i**), respectively. **e**, **k** Quantification of BRCA1 foci per EdU-positive nucleus, in samples treated as in (**c**, **i**), respectively. **f**, **l** Quantification of BRCA1 foci per EdU-negative nucleus, in samples treated as in (**c**, **i**), respectively. All immunofluorescence quantifications were based on at least four independent biological replicates; >300 cells were analyzed per condition in each replicate. Error bars, SEM. *P < 0.05, ****P < 0.0001; unpaired two-tailed Student’s *t*-test for EdU positive cells; Mann– Whitney U test for foci.

Since the expression of proteins critical for HR may be strongly dependent on cell cycle state [36, 37], we measured the percentage of cells in S-phase after CITK depletion, using pulse-labeling with EdU. In line with previous observations [18], at 24 h after siRNAs transfection the percentage of EdU positive cells was not different between control and CITK-depleted cells (Fig. 2a, b). We thus assessed BRCA1 levels under basal conditions (i.e., in absence of exogenous DNA-damaging agents). Even in this case, BRCA1 foci per nucleus were reduced in CITK-depleted cells 24 h after siRNA transfection (Fig. 2c–f) and this occurred in both EdU-positive and EdU-negative cells (Fig. 2e, f), indicating that the reduction of BRCA1 foci was independent of proliferative status. At 48 h after siRNAs transfection, we observed a reduction of EdU positive cells in CITK -depleted samples (Fig. 2g, h), in accordance with previous reports [18]. The reduction of BRCA1-positive foci was even more prominent than at 24 h, also in proliferating cells (Fig. 2i–l). All together these data indicate that CITK is required for normal recruitment of BRCA1 at DSBs after induction of DNA damage, as well as for maintaining BRCA1 levels.

### Loss of CITK but not catalytic activity impairs BRCA1 levels

**Fig. 3:**
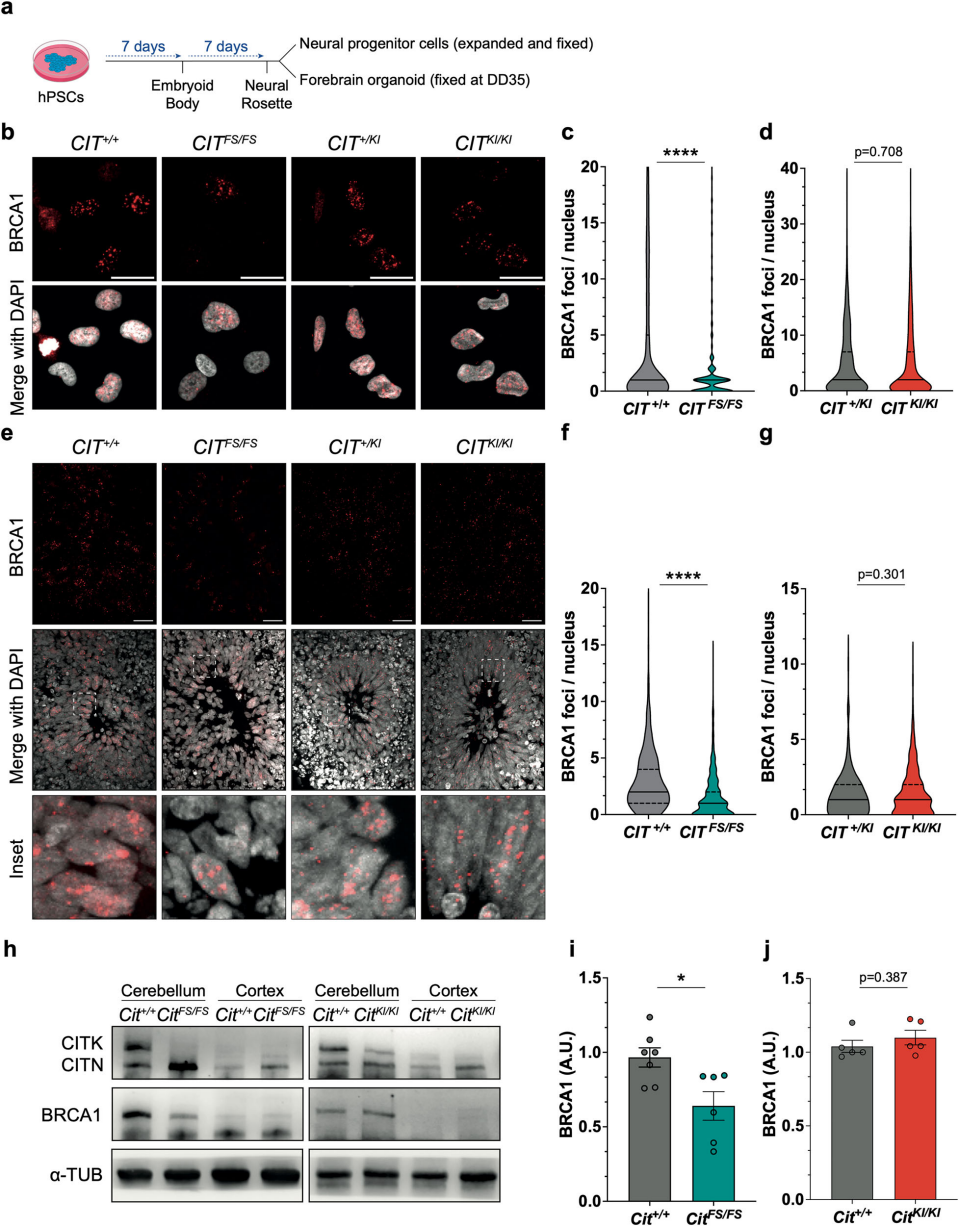
CITK scaffold activity, but not kinase activity, is required for regulation of BRCA1 levels during neurodevelopment. **a** Schematic representation of the protocol used to obtain neural progenitor cells and forebrain organoid from pluripotent stem cells. Representative images of proliferating neural progenitor cells (**b**) or rosettes from DD35 organoid sections (**e**), carrying wild type, frameshift, or kinase inactive variants of *CIT* gene, immunostained for BRCA1 and counterstained with DAPI. Scale bars: 10 μm and 50 μm, respectively. **c**, **d**, **f**, **g** Quantification of BRCA1 foci per nucleus in each genotype and condition. **h** Western blot analysis of total lysate obtained from P4 cerebellum and P4 cortex of mice carrying wild type, frameshift, or kinase inactive variant of *Cit* gene. The levels of CITK and BRCA1 were analyzed, and the internal loading control was α-tubulin (α-TUB). **i**, **j** Quantification of BRCA1 levels in each genotype. Each dot indicates an independent biological replicate. Error bars, SEM. *P < 0.05, ***P < 0.001; unpaired two-tailed Student’s *t*-test for western blots, Mann–Whitney U test for BRCA1 foci. A.U. arbitrary unit.

Biallelic clinically relevant loss of function nonsense and hypomorphic missense *CIT* variants are associated with a phenotypic range spanning from microlissencephaly to microcephaly respectively. Functionally, these genotypes correspond to a complete loss of protein or stable expression of kinase inactive CITK [11–14]. To evaluate the role of CITK catalytic activity on BRCA1-dependent functions, we took advantage of neural fated tissue differentiated from human pluripotent cells carrying clinically relevant frameshift (FS) or *CIT* kinase inactive (KI) variants. *CIT*^*FS/FS*^ human embryonic stem cells (hESCs) were obtained through CRISPR-Cas9 genome editing of the H9 hESC line, while *CIT*^*KI/KI*^ induced pluripotent stem cells (iPSCs) were obtained by reprogramming fibroblasts of individuals carrying homozygous *CIT* kinase-inactivating missense variants [38]. *CIT*^*FS/FS*^ hESCs, *CIT*^*KI/KI*^ iPSCs and their matched controls (*CIT*^*+I+*^ hESCs and *CIT*^*+/KI*^ iPSCs from a first degree relative, respectively) were differentiated into neural progenitor cells (NPCs) extracted at 14 days of differentiation (DD), and forebrain organoids fixed at 35 DD, using dual inhibition of SMAD signaling (Fig. 3a), as previously described [39]. We assessed BRCA1 levels by immunohistochemistry analysis and found that *CIT*^*FS/FS*^ NPCs showed a reduced amount of BRCA1 foci per nucleus, compared to *CIT*^*+/+*^ cells, while we did not observe significant differences between *CIT*^*KI/KI*^ and *CIT*^*+/KI*^ NPCs (Fig. 3b–d). Similarly, we found less BRCA1 foci per nucleus in DD35 neuronal rosettes of *CIT*^*FS/FS*^ organoids compared to *CIT*^*+/+*^ organoids and no differences between *CIT*^*KI/KI*^ and *CIT*^*+/KI*^ organoids (Fig. 3e–g). To determine if this phenotype is conserved, we collected *Cit* null (*Cit*^*FS/FS*^) [15] or kinase-inactivating (*Cit*^*KI/KI*^) cerebellar and cortical tissue from postnatal day (P) 4 mouse models [38]. We analyzed CITK and BRCA1 levels by western blot [40, 41]. As has been previously shown, CITK was undetectable in *Cit*^*FS/FS*^ samples and reduced to ~50% of normal value in *Cit*^*KI/KI*^ samples (Fig. 3h, i) [38]. Moreover, western blot showed a normal expression of CIT-N, a shorter brain-specific isoform lacking the amino terminal kinase domain [38]. Conversely, BRCA1 levels were significantly decreased in *Cit*^*FS/FS*^ samples but were non reduced in *Cit*^*KI/KI*^ cerebellum (Fig. 3h, i). Neither CITK or BRCA1 were expressed in P4 cerebral cortex, independent of genotype (Fig. 3h, i), consistent with the very low proliferative activity of neocortical cells at this stage [42] (Fig. 3h). Together, these data suggest that in mammals the non-catalytic structural domains of *CIT*, but not catalytic activity are required for the regulation of BRCA1 levels during development of progenitor cells.

### CITK interacts with HDAC6 and regulates its nuclear levels

**Fig. 4:**
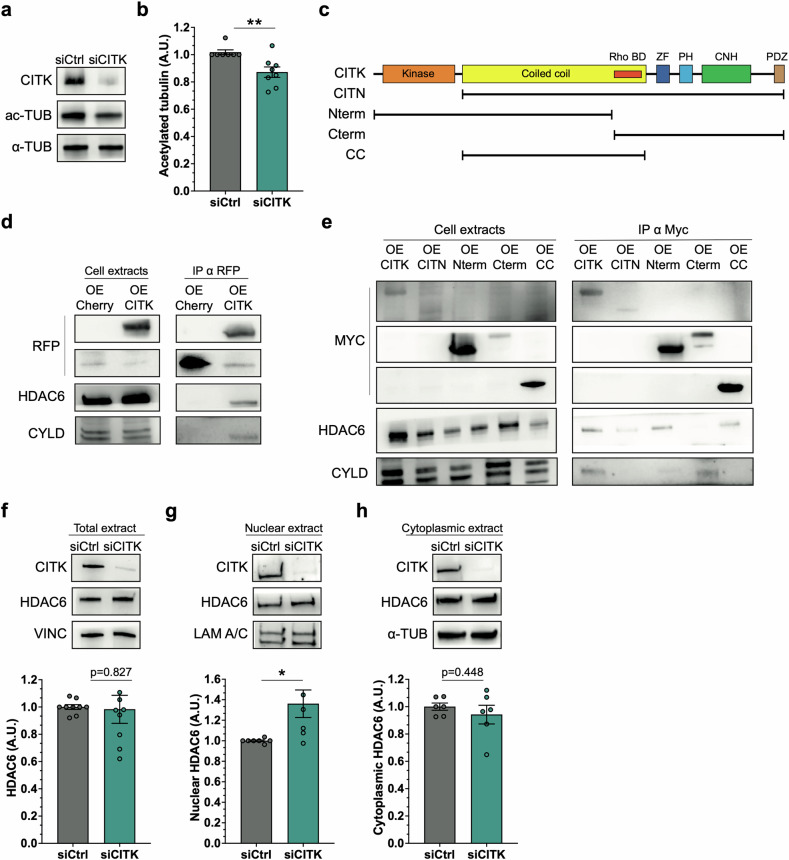
CITK regulates acetylated tubulin levels and co-immunoprecipitates with HDAC6. **a** Western blot analysis of total lysate from ONS-76 48 h after transfection of the indicated siRNA. The levels of CITK and acetylated tubulin (ac-TUB) were analyzed, and the internal loading control was α-tubulin (α-TUB). **b** Quantification of the relative density of ac-TUB in samples described in (**a**). **c** Schematic diagram of CITK protein domains (Kinase domain; Coiled coil, Rho-binding domain (Rho BD); Zinc finger (ZF), pleckstrin-homology domain (PH), Citron–Nik1 homology domain (CNH), PDZ domain) and of the different constructs used in the following panels. **d** Western blot analysis of total cell lysates (left) or RFP-Trap-based immunoprecipitations (right), obtained from 293-T cells transfected with constructs expressing Cherry-tagged CITK or with empty control vecto. The levels of RFP, HDAC6, and CYLD were analyzed. **e** Western blot analysis of total cell lysates (left) or anti-Myc immunoprecipitations (right), obtained from 293-T cells transfected with vectors expressing the indicated Myc-tagged CITK proteins. The levels of Myc, HDAC6 and CYLD were analyzed. **f** Western blot analysis of total lysate from ONS-76 48 h after transfection with the indicated siRNAs. The levels of CITK and HDAC6 were analyzed, and the internal loading control was Vinculin (VINC). Quantification of HDAC6 levels in siCITK cells relative to siCtrl. Nuclear (**g**) and cytoplasmic (**h**) fractionation of ONS-76 48 h after siRNA transfection. The levels of CITK and HDAC6 were analyzed, and the internal loading control was lamin A/C (LAM A/C) for the nucleus and α-tubulin (α-TUB) for the cytoplasm. Each dot indicates an independent biological replicate. Error bars, SEM. *P < 0.05, **P < 0.01. unpaired two-tailed Student’s *t*-test. A.U. arbitrary unit.

There is growing appreciation for the role of the microtubule cytoskeleton in nuclear recruitment of repair proteins and DNA repair during interphase [30–32]. Microtubules also form the structural basis of CITK-dependent mitotic functions [22, 23], implicating a possible role for CITK in microtubule-dependent BRCA1 and DNA DSBs repair during interphase. To test this possibility, we assessed the total levels of alpha-tubulin acetylated on lysine-40 (ac-TUB) in ONS-76 cells, as this modification has emerged as a key indicator of microtubule stability [43–45]. We found a significant reduction in ac-TUB levels in *CITK* knockdown samples, as compared to controls (Fig. 4a, b). In search for mechanisms that may connect microtubule acetylation with DSBs repair downstream of CITK, we re-analyzed a previously published CITK interactome [46]. In this dataset, CYLD, a deubiquitinating enzyme that may stabilize microtubules through histone deacetylase 6 (HDAC6) emerged as a prioritized interaction [47–49]. In the cytoplasm, HDAC6 promotes microtubule instability by de-acetylating alpha-tubulin, an activity inhibited by direct interaction with CYLD [50]. This interaction is also notable as it highlights the nuclear functions of HDAC6, which impairs recruitment of BRCA1 at DSBs, by inhibiting H2A/H2A.X ubiquitination, identifying a novel association between CITK and nuclear functions during interphase of the cell cycle [49]. To validate this interaction, we performed a co-immunoprecipitation. This analysis confirmed that endogenous CYLD and HDAC6 are consistently immunoprecipitated with recombinant CITK expressed in 293 T cells (Fig. 4c, d). To dissect these interactions, CITK constructs that included the coiled-coil region (CC) and C-terminal domain, were overexpressed and analyzed. Endogenous HDAC6 co-immunoprecipitated with the coiled-coil region (CC) but not with the C-terminal domain (Fig. 4e). On the other hand, endogenous CYLD was efficiently co-precipitated with the C-terminal domain (Fig. 4e). Next, we wondered if CITK regulates HDAC6 levels or its nuclear localization. By western blotting, HDAC6 total levels did not differ between control and CITK-depleted ONS-76 cells 48 h after siRNA transfection (Fig. 4f). However, nuclear-cytoplasmic fractionation revealed in CITK-depleted cells a tendency to lower HDAC6 cytoplasmic levels and a significant increase in HDAC6 nuclear levels (Fig. 4g, h).

### HDAC6 targeting recovers BRCA1 levels and counteracts DNA damage in CITK depleted cells

**Fig. 5:**
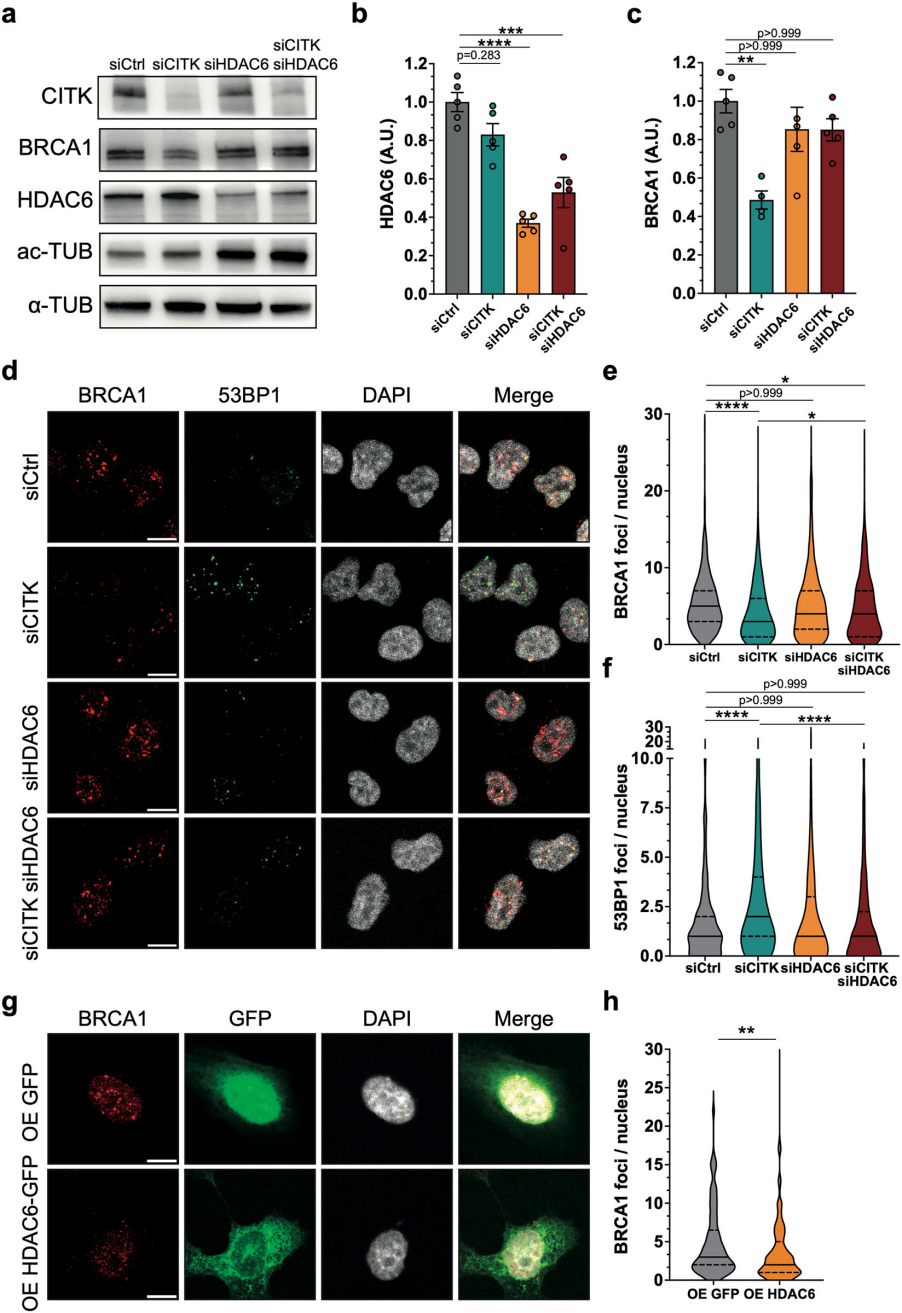
HDAC6 targeting recovers BRCA1 levels and DNA damage in CITK depleted cells. **a** Western blot analysis of total lysate from ONS-76 cells, 48 hafter transfection of the indicated siRNAs. The levels of CITK, HDAC6, acetylated tubulin (ac-TUB) were analyzed, and the internal loading control was α-tubulin (α-TUB). **b** Quantification of the relative density of HDAC6 in each treatment condition. **c** Quantification of the relative density of BRCA1 in each treatment condition. **d** Representative images of ONS-76 cells, analyzed 48 h after transfection of the indicated siRNAs. Cells were immunostained for BRCA1 and 53BP1 and counterstained with DAPI. Scale bars: 10 μm. Quantification of BRCA1 (**e**) or 53BP1 (**f***)* foci per nucleus in experiments performed as in (**d**). **g** Representative images of ONS-76 cells after transfection with GFP or HDAC6-GFP expression plasmids. Cells were immunostained for BRCA1 and counterstained with DAPI. Scale bars: 10 μm. **h** Quantification of BRCA1 foci per nucleus in samples treated as in (**e**) condition. Each dot indicates an independent biological replicate. All immunofluorescence quantifications were based on at least four independent biological replicates; >300 cells were analyzed per condition in each replicate. Error bars, SEM. **P < 0.01, ***P < 0.001, ****P < 0.0001; one-way ANOVA test followed by Bonferroni’s correction (**b**, **c**, **e**, **f**), unpaired Student’s *t*-test (h). A.U. arbitrary unit.

The previous findings that HDAC6 overexpression inhibits while nuclear HDAC6 degradation facilitates DSB repair [49] suggest that the increase of nuclear HDAC6 may mediate the effects of CITK depletion on BRCA1 recruitment and DSB accumulation. We therefore tested whether targeted reduction of HDAC6 reverses the phenotypes produced by CITK depletion. By RNAi, an approximately 50% reduction in HDAC6 levels was detected 48 h after siRNA transfection, which correlates with an ~50% increase in acetylated tubulin levels (Fig. 5a, b). In addition, HDAC6 knockdown alone did not alter BRCA1 expression compared to control conditions (Fig. 5c). In line with a functional interaction between CITK and HDAC6, in CITK–HDAC6 double knockdown samples BRCA1 abundance was rescued from reduced CITK knockdown to control levels (Fig. 5c). Accordingly, by immunofluorescence analysis, the average number of BRCA1 foci per nucleus and number of 53BP1-positive foci per nucleus were again comparable to control cells in double CITK–HDAC6 knockdown samples (Fig. 5d–f). Conversely, over-expression of GFP-tagged HDAC6 in ONS-76 resulted in a reduced amount of BRCA1 foci per nucleus, when compared to overexpression of GFP alone (Fig. 5g, h).

We next assessed the effect of modulating HDAC6 activity, using the HDAC6-specific inhibitor Tubastatin A (TubA) [47, 51, 52]. Treatment of basal ONS-76 cells with 25 nM TubA for 24 h increased the levels of acetylated tubulin without affecting by itself the levels of DNA damage or the percentage of binucleated cells, when compared to DMSO alone (Supplementary Fig. 2a–d). The same treatment, performed 24 h after transfection with anti-CITK siRNAs, reverses the CITK binucleation phenotype (Supplementary Fig. 2e–g), indicating that low doses of TubA effectively rescue the cytokinesis defect produced by CITK loss during mitosis. Interestingly, TubA treatment also led to ~20% reduction in HDAC6 expression (Supplementary Fig. 3a). In particular, nuclear-cytoplasmic fractionation revealed that this decrease preferentially impacts HDAC6 protein levels in the nucleus of TubA -treated cells, while no significant changes were detected in cytoplasmic levels (Supplementary Fig. 3b, c). TubA significantly increased BRCA1 nuclear accumulation (Supplementary Fig. 3f, g) in CITK depleted cells, as compared to cells treated with DMSO control. Importantly, TubA also restored to control levels the increase of 53BP1 foci induced by CITK loss (Supplementary Fig. 3f, h). To further assess whether this rescue may depend on microtubule stability, we performed a similar experiment (Supplementary Fig. 4a) using the microtubule stabilizing agent Paclitaxel, which at 1 nM concentration was previously found to rescue both cytokinesis failure and spindle positioning defects in CITK-depleted cells [22, 23]. As expected, paclitaxel treatment increased acetylated tubulin in both control and CITK-depleted cells (Supplementary Fig. 4b). BRCA1 nuclear foci were reduced by Paclitaxel treatment in control cells and the reduction was not worsened by CITK RNAi (Supplementary Fig. 4c, d). Conversely, 1 nM Paclitaxel did not increase the number of 53BP1-positive nuclear foci in cells transfected with control siRNAs and prevented the increase produced by CITK knockdown (Supplementary Fig. 4e). As in the case of TubA, even 1 nM Paclitaxel specifically reduced HDAC6 nuclear levels (Supplementary Fig. 4f–h). These data indicate that increased microtubule stabilization reduces HDAC6 nuclear levels and prevents the increase of DNA damage produced by CITK loss.

### HDAC6 inhibition recovers microcephaly in *cita*-loss-of-function zebrafish embryos

**Fig. 6:**
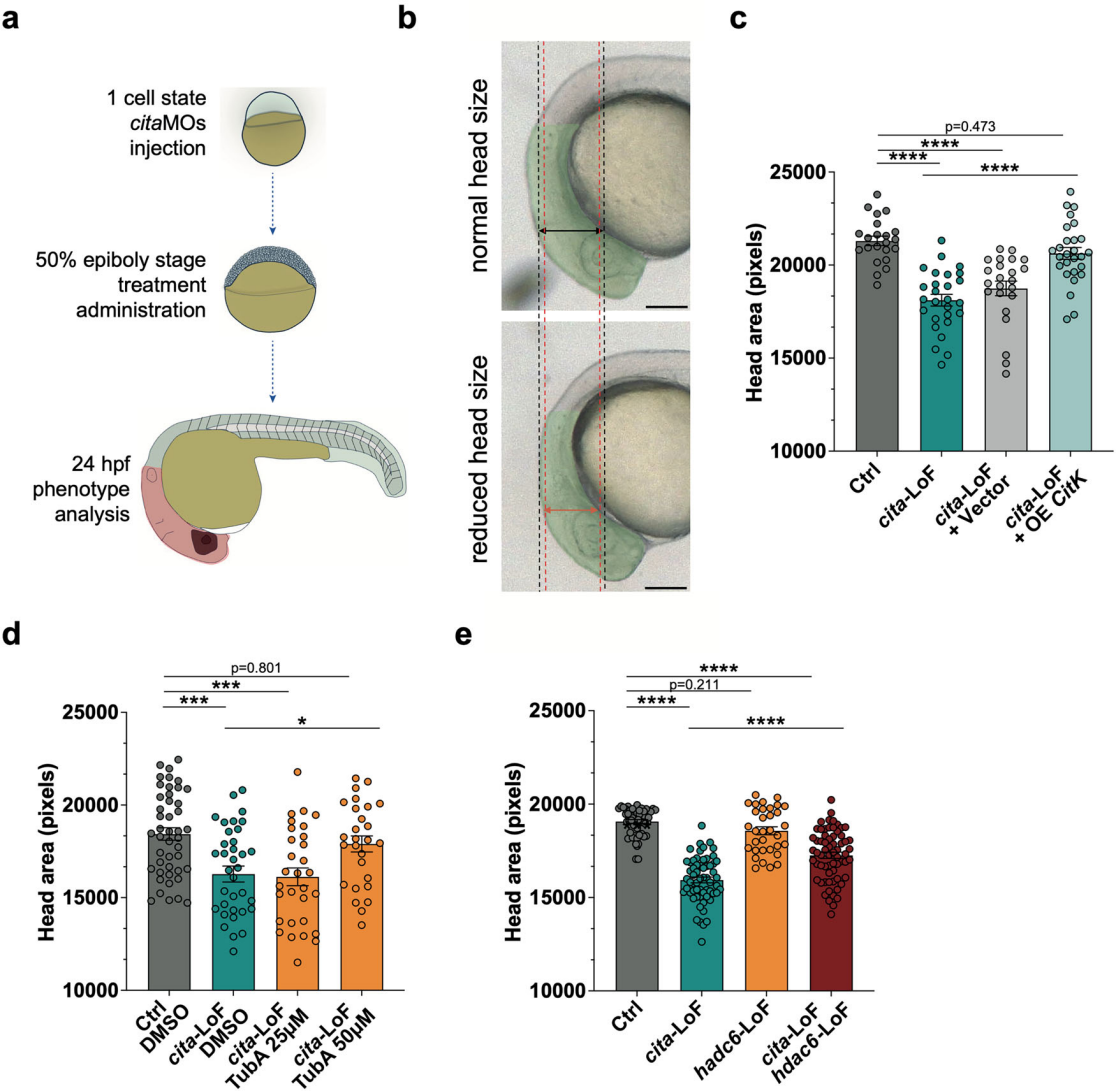
Targeting HDAC6 recovers microcephaly in MCPH17 Zebrafish model. **a** Schematic representation of the experiment performed. **b** Representative brightfield images of the head region of 24 hpf embryos showing normal or reduced head size. Scalebar = 100 μm. **c** Quantification of the lateral head area under control conditions, after injection of *cita*-LoF morpholino oligonucleotide and after co-injection of *cita*-LoF with control vector or Cit-K expression vector. **d** Quantification of the lateral head area under control conditions and in *cita*-LoF embryos treated with DMSO or Tubastin A at the indicated concentrations. **e** Quantification of the lateral head area under control condition of after injection of the indicated morpholinos. Each dot indicates a single embryo. Error bars, SEM. *P < 0.05, ***P < 0.001, ****P < 0.0001 one-way ANOVA test followed by Tukey’s correction.

Zebrafish have two *CIT* homologues, *cita* (GRCz11, Chr5:2,409,554–2,636,078) and *citb* (GRCz11, Chr8:3,820,134–3,923,378). Of the two, *cita* shows major sequence conservation with the murine and human genes at both transcript and protein levels (Supplementary Fig. 5a). Importantly, the Citb protein lacks the N-terminal region containing the kinase domain, making it more similar to CIT-N, the neuro-specific isoform not implicated in microcephaly [38]. Since *cita* is the most likely putative orthologue of CIT, we designed knockdown and transient knockout strategies to induce loss-of-function (LoF) and dissect biological roles of this gene (Supplementary Fig. 5b). Antisense morpholinos or CRISPR-Cas9 sgRNAs injection resulted in significant head size reduction without other gross morphological defects (Fig. 6a–c, Supplementary Fig. 5b–i). Subsequently, we tested whether overexpression of full-length mouse CITK (mCITK) could rescue the Cita-dependent microcephalic phenotype. Injection of 10 pg/embryo of an mCITK expression plasmid [53] significantly restored head size (Fig. 6c), underscoring the evolutionary conservation of CITK function between zebrafish and mice.

Finally, we investigated whether Hdac6 inhibition could reverse the head size reduction in *cita-*LoF embryos. We treated embryos from the 50% epiboly stage prior to neurogenesis, with two doses of TubA (25 μM and 50 μM), to take into account the possible variations in drug absorption, distribution, metabolism, and excretion between whole organisms and isolated cells. This resulted in a dose-dependent improvement in head size at 24 hpf (Fig. 6d). Similarly, knockdown of *hdac6* ameliorated the microcephalic phenotype of *cita*-LoF embryos, confirming its role in the pathogenesis of this neurodevelopmental defect (Fig. 6e).

In summary, these findings demonstrate that in zebrafish, targeting Hdac6, either through genetic manipulation or pharmacological modulation, can rescue CITK-dependent microcephaly phenotype.

## Discussion

The identification of viable therapeutic approaches for neuro-developmental disorders characterized by extensive loss of neural progenitors and neurons during development represents a very hard frontier for medicine. Indeed, the detailed studies performed on many MCPH genes have revealed that the primary defects of the encoded proteins usually impact on many intertwined cell biology mechanisms ensuring the genomic integrity of dividing neural progenitors and the correct ratio between symmetric and asymmetric cell divisions [9]. The case of MCPH17, caused by mutations in *CIT* gene, is a good example of this complexity. Total loss of CITK leads to prominent cell division abnormalities of neural progenitors including cytokinesis failure, metaphase delay/arrest and abnormal spindle positioning, which may impact on asymmetric cell divisions and differentiation timing [20, 22, 23, 53]. All these alterations could be reconciled with the capability of CITK to affect microtubules organization and dynamics in mitotic spindles and at the midbody [22]. Indeed, many of the proteins that are capable to physically and functionally interact with CITK, such as KIF23, INCENP, KIF14, ASPM, and KIF1BP, are also well known to interact with microtubules [22, 46, 54, 55]. Notably, the last three proteins are encoded by genes involved in microcephaly syndromes [56–58]. Moreover, the sensitivity of cells to CITK loss can be enhanced by conditions that increase microtubule instability, such as the expression of TUBB4 and treatment with low concentrations of microtubule-destabilizing drugs [23]. Conversely, low concentrations of the microtubule stabilizer Paclitaxel are capable to rescue both cytokinesis defects and abnormal spindle orientation in CITK-depleted cells [22]. In agreement with these observations, we here confirmed that the knockdown of CITK leads to decreased levels of acetylated alpha-tubulin, probably reflecting increased microtubule instability (Fig. 4a). However, CITK loss also leads to massive apoptosis of neural and glial progenitors, as well as of differentiated neurons [15, 59]. This phenotype correlates with accumulation of DNA DSBs, which may result from reduced RAD51 function and functional alteration of HR, leading to TP53 activation [15]. *CITK/TP53* double knockout mice display a strongly attenuated phenotype, if compared to *CITK* knockout mice, with total rescue of perinatal lethality and partial rescue of movement disorders and microcephaly [15]. Nevertheless, the pharmacological inactivation of TP53 cannot be considered a promising therapeutic approach, not only for its potential tumor-promoting effects but also because the CITK phenotype clearly involves a significant TP53-independent component [15, 18]. Thus, potential treatments for MCPH17 should be aimed at reverting both the microtubule-dependent events and the DNA damage caused by CITK loss. Since microtubule dynamics have been implicated in DNA damage repair [32], a crucial unresolved issue to address this problem was whether and how the alterations of the HR pathway produced by CITK may also involve microtubule-dependent mechanisms.

With this question in mind, we assessed the consequences of CITK loss on events preceding the recruitment of RAD51 to recessed ends, after the induction of exogenous DSBs. We found that CITK loss affects the association of BRCA1 with DSB as well as BRCA1 protein and mRNA expression (Fig. 1). The latter effect is clearly detectable only at late times of CITK knockdown, so we cannot exclude that it is an indirect consequence of CITK silencing. Indeed, 48 h after siRNA transfection, ONS-76 cells show a significant reduction in proliferation (Fig. 2) and BRCA1 mRNA and protein levels are well known to change in a cell cycle-dependent manner [60]. In contrast, the reduced recruitment of BRCA1 to DNA damage foci is an earlier event, occurring when proliferative activity and total BRCA1 levels are not significantly affected (Fig. 1). The impairment of BRCA1 levels is also present when cells are not challenged with external causes of DNA damage. Moreover, its independence from cell cycle state is supported by the fact that it is detectable in cells positive or negative for EdU incorporation after pulse labeling (Fig. 2). Although BRCA1 is heavily phosphorylated in proliferating cells [60], its recruitment to DSB is mostly independent from CITK catalytic activity, since it is not significantly affected in proliferating neural progenitors derived from MCPH17 patients with KI mutations, while it is compromised by CITK mutations leading to total protein loss (Fig. 3b–g). Similarly, BRCA1 protein levels do not change in proliferating cerebellum of mice homozygous for a KI mutation, while they are significantly reduced in CITK knockout mice (Fig. 3h–j). On this basis, we conclude that the action of CITK on BRCA1 involves structural regions located downstream of the kinase domain.

Identification of the deubiquitinating enzyme CYLD in a previously published CITK interactome [46], which we further validated here (Fig. 4) provided us with an attractive possibility to bridge CITK, microtubule stability, and BRCA1 through the involvement of alpha-tubulin deacetylase HDAC6. Indeed, CYLD binds to alpha tubulin and polymerized microtubules and increases microtubule acetylation by inhibiting HDAC6 through direct binding [50], suggesting that even HDAC6 could be part of some CITK complexes. Accordingly, endogenous HDAC6 was co-precipitated with overexpressed full length CITK, as well as with CITK mutants possessing its extended coiled-coil region, but not with the carboxy-terminal region of the protein. Conversely, CYLD interacted more strongly with the latter domain (Fig. 4d, e).

Based on these data, we could speculate that CITK may limit the deacetylation of alpha-tubulin in the cytoplasm by trapping HDAC6 in a complex containing CYLD. Although this scenario would have obvious implications for the phenotypes of CITK loss already ascribed to microtubule instability, the druggability of HDAC6 prompted us to address its functional involvement in the DNA damage-related phenotypes. HDAC6 is known to inhibit mismatch repair (MMR) by deacetylating MLH1 and MSH2 [61, 62], as well as nucleotide excision repair (NER) by deacetylating replication protein A (RPA1), thus disrupting its interaction with the critical NER factor XPA [63, 64]. Moreover, HDAC6 has recently been found to modulate DSB, by preventing the recruitment of BRCA1 through protection of H2A/H2A.X from degradation [49].

We found that knockdown of HDAC6 in cells depleted of CITK partially recovered the number of BRCA1 nuclear foci and restored to control levels 53BP1 foci (Fig. 5a–f). Similar effects were obtained by treating CITK-depleted ONS-76 cells with the HDAC6-specific inhibitor TubA (Supplementary Fig. 3) and with the microtubule stabilizing agent Paclitaxel (Supplementary Fig. 4), at concentrations that significantly increase alpha-tubulin acetylation without inducing an increase of DNA damage. Conversely, overexpression of HDAC6 decreased the number of BRCA1 nuclear foci (Fig. 5g, h). The effectiveness of TubA is in apparent contrast with the proposed mechanism of HDAC6 action on BRCA1, which was shown to depend on HDAC6 association with chromatin, but not on HDAC6 catalytic activity [49]. However, we also found that CITK knockdown specifically increases HDAC6 nuclear levels, while TubA reduces them. Based on these results and on the previous finding that HDAC6 inhibition increases its association with microtubules [65], a possible scenario is that CITK may prevent HDAC6 nuclear accumulation by recruiting it in an inhibitory complex with CYLD, which may increase its interaction with microtubules.

Although further investigation is necessary to test the mechanistic details of this hypothesis, an important conclusion of our study is that specific inhibition of HDAC6 can revert the DNA DSB accumulation produced by CITK loss, besides having the potential to directly counteract mitotic phenotypes. We tested the translational implications of this finding by generating the first Zebrafish model of MCPH17. In particular, we abolished the function of *CIT* orthologue *cita* via injection of a splice site-directed morpholino-RNAs, whose specificity was proven using a translation start site-directed sequence as well as by CRISPR/Cas9-dependent inactivation (Supplementary Fig. 5). All these models displayed a clear and specific microcephaly phenotype, which was also reverted by expression of the recombinant mouse sequence, further proving the great phylogenetic conservation of CITK (Fig. 6). Importantly, the phenotype was corrected both by knocking down HDAC6 levels and by treating the morphants with TubA (Fig. 6). The latter finding strongly support further investigation of the therapeutic potential of HDAC6 inhibitors in mammalian MCPH17 models.

## Materials and methods

### Cell culture

ONS-76 cells were cultured in RPMI medium (Euroclone, Milan, Italy) supplemented with 10% FBS (Thermo Fisher Scientific Gibco, Waltham, MA) and 1% penicillin/streptomycin (Gibco). HEK293T cells were cultured in DMEM supplemented with 10% fetal calf serum and 1% penicillin/streptomycin (Life Technologies, Thermo Fisher Scientific, Waltham, MA, USA). All cells were grown at 37 ^◦^C, in a humidified incubator, with 5% CO_2_. Cells were not recently authenticated by STR analysis but were routinely analyzed for morphological features and tested for mycoplasma contamination with the following oligonucleotide sequences: Myco1, 5′ ACTCCTACGGGAGGCAGCAGTA-3′;Myco2,5′-TGCACCATCTGTCACTCTGTTAACCTC-3′.

### *Transfection of* siRNAs and plasmids

To knockdown CITK expression, we used previously published CITK-specific siRNAs [17, 66]. As control sequence we used ON-TARGETplus non-targeting siRNAs #1 (Dharmacon, Lafayette, CO, USA). To knockdown HDAC6 we used ON-TARGETplus SMARTpool siRNAs (Dharmacon, Lafayette, CO, USA). Cells were plated on 6-well plates and transfected using 6.25 μL of the required siRNA (20 μM), together with 1.5 μL of Lipofectamine 2000 (Invitrogen, Carlsbad, CA, USA) according to the manufacturer’s instructions. CITK Cherry-tagged and Myc-tagged constructs used in this article were previously published [53]. The pm-GFP-N1 plasmid (Clontech, Mountain View, CA) was used as control and the pEGFP.N1-HDAC6 was purchased from Addgene (https://www.addgene.org). For overexpression, cells were plated on 24-well plates and transfected using 0.5 μg of DNA, together with 1.5 μL of Transit-it x1 (Mirus BIO LLC, Madison, WI, USA) according to the manufacturer’s instructions.

### Drug treatments

The following drugs were used on ONS-76 cells: Bleomycin (NSC125066) sulfate (S1214 Selleckchem, Houston, TX) at final concentration of 5 μg/ml for 1 h; Paclitaxel (S1150, Selleckchem,) at final concentration of 1 nM for 24 h; Tubastatin A (S8049 Selleckchem) at final concentration of 25 nM for 24 h.

### EdU labeling

Proliferating cells were labeled by 30 min incubation of 10 μM EdU, according to manufacturer instructions (Click-iT™ EdU, C10337, Invitrogen™).

### Antibodies

The following antibodies were used: mouse monoclonal anti-citron (#611377; Transduction Laboratories, BD Biosciences, Franklin Lakes, NJ, USA), mouse monoclonal anti-BRCA1 (D-9) (#sc-6954, Santa Cruz, Dallas, TX, USA) to detect BRCA1 from human samples, mouse monoclonal anti-BRCA1 (#sc-135732, Santa Cruz) to detect BRCA1 in from mouse samples, mouse monoclonal anti-α-tubulin (#T5168; Sigma-Aldrich, St. Louis, MO), rabbit polyclonal anti-53BP1 (#ab36823, Abcam, Cambridge, MA, USA), rabbit polyclonal anti-RAD51 (#sc-8349, Santa Cruz), rabbit polyclonal anti-γ-H2AX (S139; 20E3; #2577; Cell Signaling Technology, Danvers, DA, USA), rabbit polyclonal anti-HDAC6 (#7612S; Cell Signaling Technology), rabbit polyclonal anti-RPA32 (#ab87277; Abcam), mouse monoclonal anti-Lamin A/C (SAB4200236; Abcam), rabbit polyclonal anti-CYLD (AB-83743, Immunological Sciences, Rome, Italy).

### Immunoprecipitation and Western Blotting

For all immunoprecipitations, cells were extracted with mild lysis buffer (150 mM NaCl, 1 mM MgCl2, 50 mM Tris (pH 7), 1% NP40, 5% glycerol) together with protease inhibitors (Roche, Basel, Switzerland). Antibodies, according to the manufacturer’s protocols, and 7 μl of Dynabeads protein G (10003D, Invitrogen) were added to 1 mg of cleared lysates, and incubated for 2 h at 4 °C. RFP-TRAP affinity resin from Chromotek (rtma-10 Chromotek, Planegg, Germany) was used according to the manufacturer’s instructions. Pellets were washed four times with lysis buffer and analyzed by SDS-PAGE. For western blot, cells were lysed in RIPA buffer (1% NP40, 150 mmol/L of NaCl, 50 mmol/L of Tris-HCl pH 8, 5 mmol/L of EDTA, 0.01% SDS, 0.005% sodium deoxycholate, Roche protease inhibitors and PMSF) for 10 min at 4 °C and centrifuged at 13,000 × *g* at 4 °C for 10 min. Tissues were lysed in RIPA buffer and homogenized in the same buffer with pellet pestle (Z359971 Sigma-Aldrich) before centrifuging. For nuclear-cytoplasmic fractionation cells were lysed for 10 min on ice with the cytoplasmic extract buffer (10 mM HEPES, 60 mM KCl, 1 mM EDTA, 0.1% (v/v) NP-40, 1 mM DTT, Roche protease inhibitors and PMSF). Samples were centrifuged at 1500 rpm for 5 min. Cytoplasmic extract was collected. Pellet was washed and incubated with nuclear extract buffer (20 mM Tris Cl, 420 mM NaCl, 1.5 mM MgCl2, 0.2 mM EDTA, PMSF and 25% (v/v) glycerol), after vortexing, for 30 min on ice. Samples were centrifuged at 1500 rpm for 5 min. Nuclear extract was collected. For immunoblots, equal amounts of were resolved by SDS–PAGE and blotted to nitrocellulose membrane.

### Quantitative RT-PCR

Total RNA was extracted with the RNeasy micro kit (Qiagen GmbH, Hilden, DE), and reverse transcribed to cDNA with the High-Capacity cDNA Archive kit (Applied Biosystems, Thermofisher, Waltham, USA). Quantitative Real Time RT-PCR was performed as described in [16] by combining the RealTime Ready Universal Probe Library (UPL, Roche Diagnostics, Monza, Italy) with the primers listed in the Supplementary Table 1. Data was collected on the Applied Biosystems StepOnePlus Real-Time PCR System with StepOne™ Software. A relative quantification approach was used, according to the 2-ddCT method. β-actin was used to normalize expression levels.

### Immunofluorescence

Cells were fixed for 10 min at RT with PFA 2%, treated for 10 min at RT using CSK buffer (100 mmol/L of NaCl, 300 mmol/L of sucrose, 3 mmol/L of MgCl2, 10 mmol/L of PIPES (pH 6.8) and 0.7% Triton), and fixed again, for 5 min, at RT, using PFA 2%. Subsequently, cells were permeabilized with 0.5% Triton X-100 in PBS for 20 min, saturated in 5% BSA in PBS for 30 min, and incubated with a primary antibody for 2 h at RT. Primary antibodies were detected with anti-rabbit or anti-mouse Alexa Fluor 488, 555, 647 (Thermo Fisher Scientific) at 1:1000 dilution for 30 min. Cells were counterstained with 0, 5 mg/mL of DAPI for 10 min and washed with PBS. Finally, cell slides were mounted with Prolong (Thermo Fisher Scientific). Click-iT™ EdU (C10337, Invitrogen™) was performed according to manufacturer instructions.

### hPSC culture

Human pluripotent and embryonic stem cells were obtained and generated as described in [38]. Cells were maintained in mTeSR1 medium (#85850, StemCell Technologies, Vancouver, Canada) on Matrigel-coated dishes (#354234, Corning, Corning, NY) in a cell incubator at 37 °C with 5% CO2. Stem cells were chemically dissociated at 37 °C using Versene (#15040066, Invitrogen) and then resuspended in mTeSR1 with added Y27632 ROCK inhibitor (Cat. No. 1254 Tocris, Bristol, UK) at a final concentration of 10 μM for 24 h to promote survival when cells were passaged. mTeSR1 medium was changed daily.

### hPSCs differentiation

hPSCs were differentiated to NPCs and dorsal forebrain organoids using dual inhibition of SMAD signaling as previously described [39]. Briefly, stem cells were chemically dissociated at 37 °C using StemPro^TM^ Accutase^TM^ Cell Dissociation Reagent (#A1110501, Gibco) and resuspended in mTeSR1 with added Y27632 ROCK inhibitor at a final concentration of 10 μM. Cells were counted and ~650 cells were plated in ultra-low cell attachment 96-well plate with a V-shaped bottom (#MS- 9096VZ S-Bio, Hudson, NH) in a final volume of 30 μL. This step is considered day 0 of differentiation (0DD). Neural induction was initiated 36 h later (1.5DD) by adding 150 μL of N2 + dual SMAD inhibition medium (1:100 N2 supplement (#17502048 ThermoFisher), 1 μM Dorsomorphin dihydrochloride (Cat. No. 3093 Tocris), 2 μM A83-01 (Cat. No. 2939 Tocris) in DMEM/F12 (#11320033 Gibco) to each well. Media was changed every 48 hours. On 7DD, medium was changed to N2 + B27 + Dorsomorphin medium (1:200 N2 supplement, 1:100 B27 supplement (#17504044 ThermoFisher), 1 μM Dorsomorphin, 20 ng/mL bFGF (100-18B-100UG ThermoFisher) in DMEM/F12) and aggregates were transferred to a 6-well plate coated with Matrigel using a 200 μL pipette with a razor cut tip to minimize shearing forces on the aggregates (~12–15 aggregates per well). Extra media was then added to reach 2 mL of N2 + B27 + Dorsomorphin per well. Media was changed every 48 h. On 14DD, NPCs were extracted by gently detaching neural rosettes from Matrigel using a p200 tip, followed by chemical dissociation using Versene. Dissociated cells were plated on 15 μg/mL poly-L-ornithine (P4957-50ML Sigma) and 10 μg/mL laminin (L2020-1MG Sigma) coated dishes. For dorsal forebrain organoid generation, neural rosettes were gently detached from Matrigel using a p200 tip and the neural tissue was grown in suspension under constant rotation at 95 rpm. The N2 + B27 medium (1:200 N2 supplement, 1:100 B27 supplement, 20 ng/mL bFGF in DMEM/F12) was used, with medium change from 14DD to 35DD every other day.

### Tissue cryopreservation, embedding, and sectioning

Organoids were fixed in 4% PFA overnight before beginning cryopreservation. Organoids were saturated in a 15% sucrose solution overnight or until tissue sank to the bottom of the conical tube before applying the final 30% sucrose solution. Once tissue was sufficiently cryopreserved, the organoids were placed in a plastic cassette containing OCT medium (Fisher Brand) and frozen using −60 °C to −70 °C 2-methyl butane. OCT-containing organoid blocks were sectioned at 13μm using a cryostat (Leica Biosystems, Wetzlar, Germany).

### Immunofluorescence on Organoids

13 μm cryopreserved cerebral organoid sections on slides (Fisher Brand) were washed three times with 1xPBS for five minutes. Tissue was then permeabilized with 0.1% Triton X-100 for five minutes, and subsequently washed once with 1xPBS for five minutes before blocking buffer was implemented. Organoid sections were incubated with blocking buffer (5.1 mL 0.1% Triton X-100, 600 μl 10% BSA, and 300 μl NDS) for one-hour at room temperature. Primary antibodies were diluted in blocking buffer and incubated at 4 °C overnight. Sections were then exposed for 1–2 h at room temperature to secondary anti-donkey antibodies conjugated with Alexa- Fluor (Invitrogen) and finally incubated with Hoechst (Invitrogen) diluted at 1:10,000 in PBS for five minutes. Coverslips were mounted on the glass slides using Prolong Gold (Invitrogen) overnight before imaging.

### Mouse strains

The mouse strain containing the frameshift *Cit* loss of function variant (Cit^FS/FS^) in a C57BL/6 background was obtained from UC Davis KOMP repository (*Cit* ^*tm1a(KOMP)Wtsi*^). The mouse strain containing Cit kinase inactive mutation was generated as described in [38]. Briefly, we mutagenized *Cit* (c.376_377AA > GC; p.K126A) to generate K126A substitution in the catalytic pocket in exon 3. This substitution totally inactivates the kinase domain and resembles pathogenic *CIT* variants found in affected individuals [12]. Heterozygous (*Cit*^+/KI^) mice obtained from germline transmission were intercrossed to generate homozygous (*Cit*^KI/KI^) and control (*Cit*^+/+^) progeny. Age-matched wild-type littermates were used as controls.

### Zebrafish’s husbandry

Adult AB wild-type (European Zebrafish Resource Center, EZRC) zebrafish were maintained following national guidelines (Italian decree March 4, 2014, n. 26) at 28 °C and subjected to a 14-h light and 10-h dark cycle. Fertilized eggs, obtained by breeding pairs, were collected, transferred to fresh E3 medium (50X stock solution: 73.0 g of NaCl, 3.15 g of KCl, 9.15 g of CaCl2, and 9.95 g of MgSO4 in 5 L of distilled H2O) and maintained at 28 °C for up to the desired developmental stage. From 24 hpf (hours post fertilization) onwards, the E3 medium was supplemented with 0.003% 1-phenyl-2-thiourea (PTU, Sigma-Aldrich, Saint Louis, MO) to prevent pigmentation. The chorions were mechanically removed using thin needles or tweezers and then anesthetized with a 0.016% tricaine solution (ethyl 3-aminobenzoate methanesulfonate salt; Sigma-Aldrich) as described in [67].

### Bioinformatic analyses for CIT zebrafish orthologue

CIT transcripts and protein sequences (Ensembl) were analyzed using the pairwise alignments Needle-EMBOSS tool (Clustal, EMBL-EBI) [68]. The reference codes of the sequences used for the analyses are listed in the Supplementary Table 2.

### Knock-down, transient knock-out and rescue experiments in zebrafish

*cita* and of *hdac6* knock-down was achieved using morpholino (MOs) oligonucleotides obtained from Gene Tools (Philomath, OR, USA). *cita*-ATG-MO (ATATTTAACTTCAACATCACTGCAGG) and *cita*-sMO (CACTTCCCTGGTGAAACACAAAATA) were used for single (0.0625 pmol/embryo) or combined (0.125 pmol/embryos) injection into 1-2 cell embryos (Supplementary Table 3). For *hdac6* knockdown we used a previously published *hdac6*-MO [69] selecting 0.0625 pmol/embryo as working dose. Control embryos were injected with standard (std) MO (CCTCTTACCTCAGTTACAATTTATA). *cita* splicing morpholino (*cita*-sMO) was validated by RT-PCR starting from total RNA of 24 hpf control and *cita*-sMO injected embryos (~30 embryos per condition) using the NucleoZOL one-phase RNA purification reagent according to the manufacturer’s instructions (Macherey-Nagel Düren, Germany) as described in [70]. After DNase tratement with RQ1 RNase-Free DNase kit (Promega, Madison, WI, USA) cDNA was synthetyzed using the GoScript™ Reverse Transcriptase cDNA Synthesis kit (Promega, Madison, WI, USA). β-actin and *cita* primers (Supplementary Table 4) designed with the Open Source Primer3 Software were used to generate PCR amplicons (GoTaq® G2 DNA Polymerase, Promega, Madison, WI, USA). The PCR bands were isolated from a 2% agarose gel, purified, and subjected to Sanger sequencing (Wizard® SV Gel and PCR Clean-Up SystemPromega).

CRISPR-Cas9 20-base pair crRNAs specific to *S. pyogenes* Cas9 (SpCas9) were designed with the CRISPOR web tool (http://crispor.tefor.net) [71] using the GRCz11 zebrafish genome assembly as reference (Supplementary Table 5). sgRNAs were purchased by IDT as Alt-R CRISPR-Cas9 crRNA (2 nmol/ml) and Alt-R CRISPR-Cas9 tracrRNA (5 nmol/ml) and used following manufacturer’s instructions. 1 μl of each crRNA was combined with 1 μl of tracrRNA, incubated at 95 °C for 5 min, cooled to room temperature, and supplemented with 0.2 μl of 10X CRISPR buffer (20 mM Hepes-NaOH pH 7.5, 0.15 M KCl). After mixing 0.5 μl of each crRNA/tracrRNA solution and 1 μl of Alt R S.p. Cas9 nuclease V3 (10 μg/μl) protein (IDT), 3 nl of the injection were micro-injected into one-cell stage zebrafish embryos.

For rescue experiments, 10 pg/embryo of the expression construct coding for full-length mouse Myc-Cit-K [53] were co-injected with *cita*-MOs at 1–2 cell stage. As a control, embryos were injected with the same amount of the empty vector as previously described [53].

### T7 Endonuclease I assay

The T7 endonuclease I assay employs a mismatch-specific DNA endonuclease (T7E1) to identify the mutations in a target DNA region. This process involves PCR amplification of the targeted region, followed by digestion with specific restriction enzymes using the EnGen® Mutation Detection Kit (New England Biolabs, Ipswich, UK). Variations in the sizes of digested and undigested PCR fragments were analyzed using agarose gel electrophoresis.

### Chemical treatments in zebrafish embryos

TubastatinA (TubA; Sigma) was dissolved in Dimethyl sulfoxide (DMSO) was administrated directly in the E3 medium at a final concentration of 25 or 50 μM as previously described [69]. Pharmacological treatments were performed in a 24 multi-well plates placing a maximum of 15 embryos/well from the stage of 50% epiboly (6 hpf) to 24 hpf.

### Zebrafish head size measurement

24 hpf embryos images were acquired under Leica stereomicroscope equipped with a digital camera and LAS Leica Imaging software version 4.13 (Leica). Images were taken from a lateral and a dorsal view and areas were measured using Fiji (ImageJ) software selecting a region of interest (ROI).

### Study approval

For iPSC lines, subjects were enrolled according to protocols approved by institutional review boards at affiliated institutions, as previously described [11]. In all cases, the procedures followed were in accordance with the ethical standards of the respective institution’s committee on human research and were in keeping with international standards. Written informed consent was obtained prior to participation. The mouse studies were designed according to the guidelines of the NIH, the European Communities Council (2010/63/EU) and the Italian Law for Care and Use of Experimental Animals (DL26/2014), under permission number 1128/2020-PR, released on 16th November 2020 from Italian Ministry of Health, Department of Public Veterinary Health. It was also approved by the Italian Ministry of Health and the Bioethical Committee of the University of Turin. Zebrafish were bred following international (EU Directive 2010/63/EU; European recommendations) and national recognized guidelines which focus on the protection of animals used for scientific research. Zebrafish embryos/larvae were raised for a maximum of 120 h post-fertilization, which is the developmental stage limit for excluding zebrafish from being considered an animal model (Italian Decree of March 4, 2014, n. 26).

### Statistics and general methods

Statistical analyses were performed by using Microsoft Office Excel and GraphPad (Version 10, GraphPad Software, San Diego, CA, USA). Data are shown as the mean values of at least 3 independent experiments and standard error of the mean (mean ± SEM). Mann–Whitney test was used to analyze BRCA1 and 53BP1 foci. Unpaired two-tailed Student’s *t*-test was used to compare two groups and One-way ANOVA test were used for multiple group comparisons followed by Bonferroni’s or Tukey’s correction. The number of independent biological replicates and the sample size of each replicate are indicated in the figure legends. For experiments performed on mouse samples or Zebrafish embryos, the statistical power was set to at least 90%, by distinguishing random variations (±15%) from genotype or siRNA effect (20%) at a significance level of p = 0.05. The study was conducted on subsequent samples, with correct genotype as the only criterion of inclusion in groups, and no exclusion criteria. No randomization and no blinding procedures were adopted.

## Supplementary information

Supplementary information

Unedited gels

## Data Availability

The original contributions presented in the study are included in the article and supplementary material. Further inquiries can be directed to the corresponding authors.
