# Supplementary material for: CITK modulates BRCA1 recruitment at DNA double strand breaks sites through HDAC6
Source: Cell Death Dis. 2025 Apr 20;16(1):320. doi: 10.1038/s41419-025-07655-4 (PMC12009987; doi:10.1038/s41419-025-07655-4)
Supplement: Supplementary file 1 — Supplementary information [file 41419_2025_7655_MOESM1_ESM.pdf]

Supplementary data for

CITK modulates BRCA1 recruitment at DNA double strand breaks sites through HDAC6

Giorgia Iegiani[1,2*], Gianmarco Pallavicini[1,2*], Alex Pezzotta[3], Alessia Brix[3], Alessia Ferraro[1,2], Marta Gai[4], Enrica Boda[1,2], Stephanie L. Bielas[5,6,7], Anna Pistocchi[3], Ferdinando Di Cunto[$1,2]

1. Neuroscience Institute Cavalieri Ottolenghi, Turin, Italy.

2. Department of Neuroscience 'Rita Levi Montalcini', University of Turin, Italy

3. Department of Medical Biotechnology and Translational Medicine, University of Milan, Italy

4. Department of Molecular Biotechnology and Health Sciences, University of Turin, Italy

5. Department of Human Genetics, University of Michigan Medical School, Ann Arbor, MI, USA

6. Neuroscience Graduate Program, University of Michigan Medical School, Ann Arbor, MI, USA

7.Department of Pediatrics, University of Michigan Medical School, Ann Arbor, MI, USA

* These authors equally contributed to the manuscript.

$ Corresponding author:

Ferdinando Di Cunto (Ferdinando Di Cunto, Neuroscience Institute Cavalieri Ottolenghi Regione Gonzole, 10, 10043 Orbassano (TO), Italy, +39-011-6706616, ferdinando.dicunto@unito.it)

The authors have declared that no conflict of interest exists.

Supplementary Table 1

Primer name	FW Sequence	RV Sequence	UPL probe
BRCA1	GGGTGTTGGACAGTGTAGCA	TGGGGGATCTGGGGTATCAG,	probe UPL#: 17
RAD51	AACCTTAAGTGCTGCAGCCT	GTCACAACAGGAAGAGGCCT	probe UPL#: 32
BACT	TCAACACCCCAGCCATGTAC	ATCACGATGCCAGTGGTACG	probe UPL#: 64

Supplementary Table 2

Gene	Species	Transcript ID	UniProt Match
**CIT**	human	ENST00000392521.7 (NM_001206999.2)	O14578-4
Cit	mouse	ENSMUST00000102560.7	D3YU89
cita	zebrafish	ENSDART00000122274.3	E7FGD8
citb	zebrafish	ENSDART00000122454.3	F1QN82

Supplementary Table 3

<table>
<tr><th>Morpholino</th><th>Targeted region</th><th>Sequence 5’-3’</th><th colspan="2">Dose injected per embryo</th></tr>
<tr><td>cita-ATGMO</td><td>ATG region</td><td>ATATTTAAACTTCAACATCACTGCAGG</td><td>0.0625 pmol</td><td rowspan="2">0.125 pmol total</td></tr>
<tr><td>cita-sMO</td><td>Intron1-exon2</td><td>CACTTCCCTGGTGAAACACAAAATA</td><td>0.0065 pmol</td></tr>
<tr><td>hdac6-MO</td><td>ATG region</td><td>CTTTGGTATCTGGAACCGCATCCAT</td><td colspan="2">0.00625 pmol</td></tr>
</table>

Supplementary Table 4

crRNA name	Sequence (5’-3’)
cita-crRNA1	TCGAGCGCAGCGGCTTGTGA
cita-crRNA2	TTTAAGTACCTGCCTACTAG

Supplementary Table 5

Primer name	Sequence (5’-3’)	Purpose
cita-FwdE1	CTCGACTCAATCAGCTGCTG	RT-PCR for *cita*-sMO validation
cita-RevI1	TCTACCGTTGTGCCTCCAAT	RT-PCR for *cita*-sMO validation

Supplementary images and figures

SUPPLEMENTARY FIGURE 1 | CIT-K knockdown reduces BRCA1 RNA levels and colocalization with γH2AX 48 hours after siRNA transfection

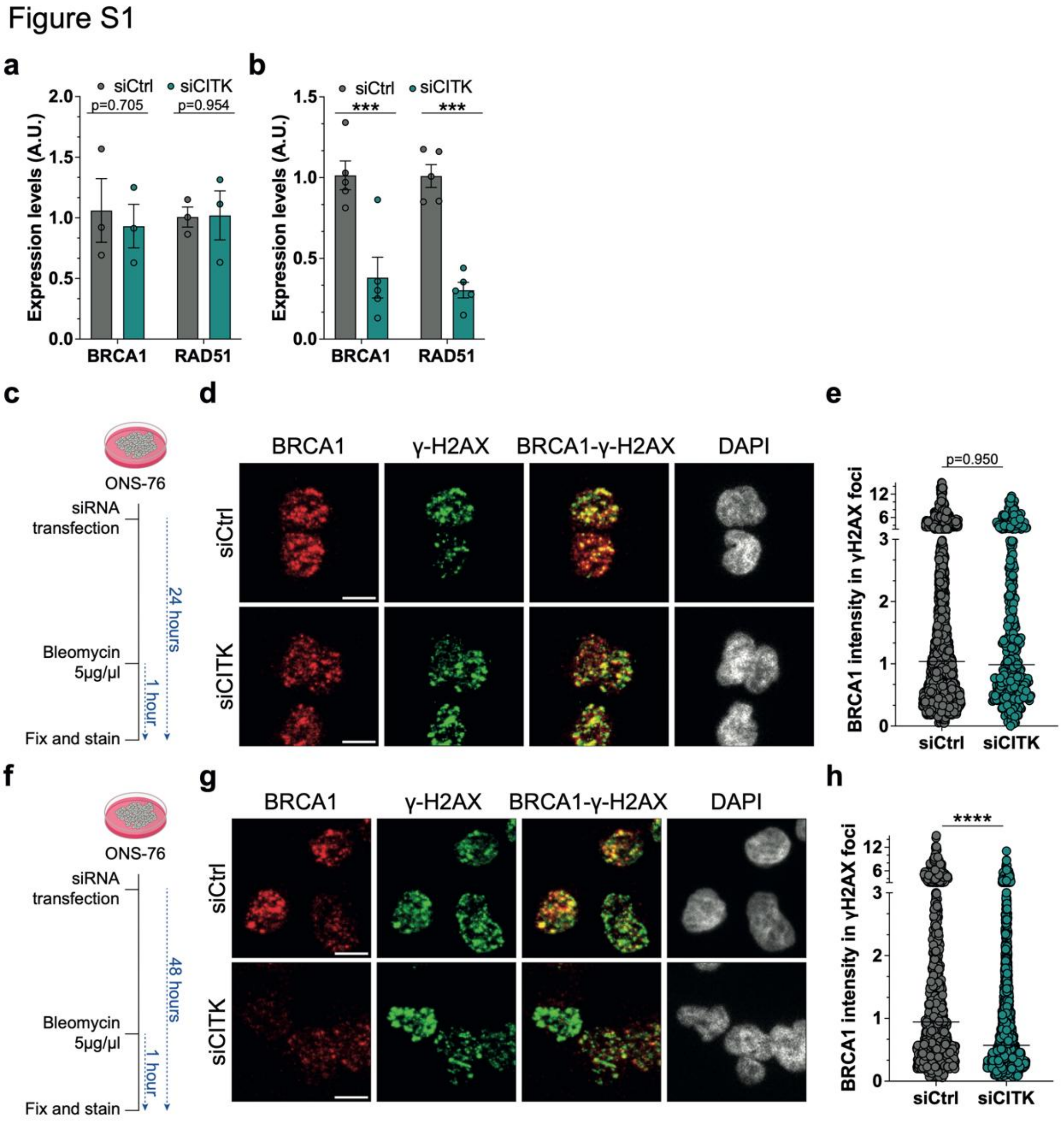

(**a-b**) Quantification of BRCA1 and RAD51 RNA levels 24 and 48 hours, respectively, after siRNA transfection. (**c, f**) Schematic representation of the experiments. (**d, g**) Representative images of ONS-76 cells treated as highlighted in (*c*) or (*f*), respectively. Cells were immunostained for BRCA1 and γH2AX and counterstained with DAPI. Scale bars: 10 μm. (**e, h**) Quantification of BRCA1 signal intensity in each 53BP1 focus. Signal was normalized to control median. Each dot indicates an independent biological replicate. All immunofluorescence quantifications were based on at least four

independent biological replicates; >300 cells were analyzed per condition in each replicate. Error bars, SEM. ***P<0.001, ****P<0.0001; unpaired two-tailed Student's t-test for RNA levels; Mann–Whitney U test for BRCA1 intensity.

SUPPLEMENTARY FIGURE 2 | Evaluation of Tubastatin A dose dependent effects on ONS-76 cells.

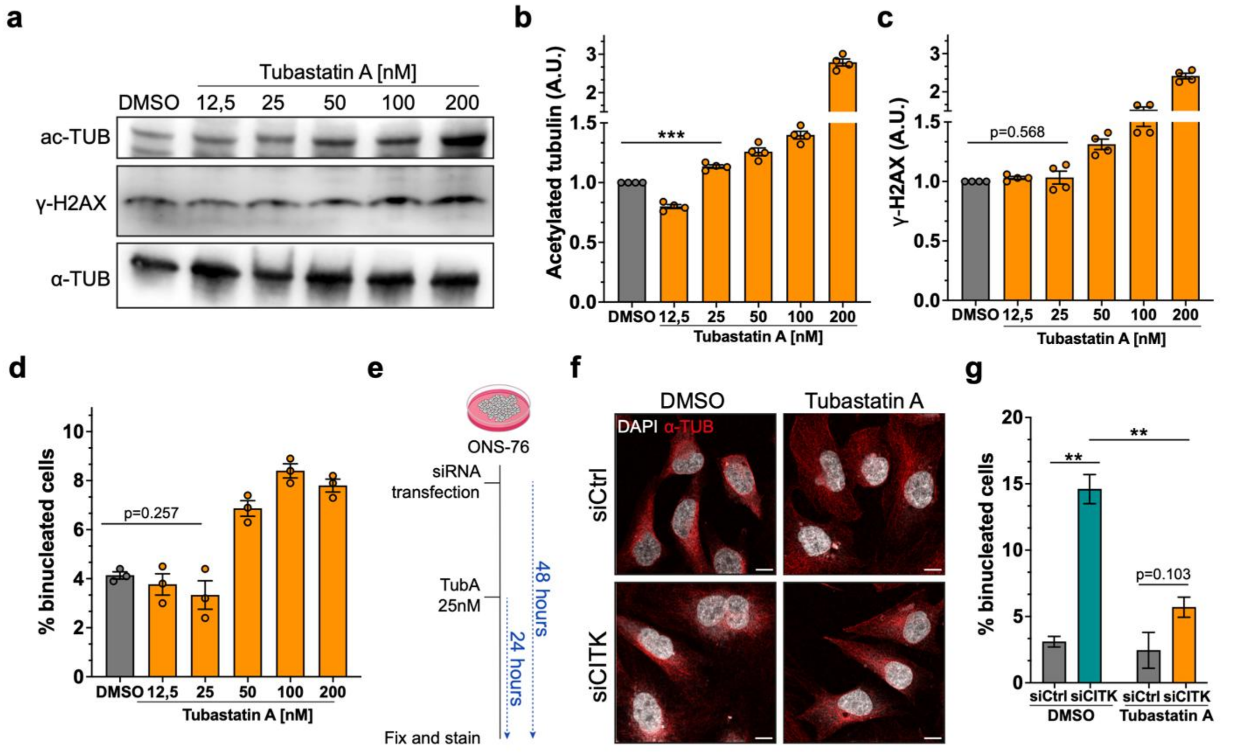

(**a**) Western blot analysis of total lysate from ONS-76, 24 hours after treatment with Tubastatin A at the indicated concentrations or vehicle (DMSO). The levels of acetylated tubulin (ac-TUB) and γ-H2AX were analyzed, and the internal loading control was α-tubulin (α-TUB). (**b-c**) Quantification of the relative density of acetylated tubulin (*b*) and γ-H2AX (*c*) in each treatment condition. (**d**) Quantification of the percentage of binucleated ONS-76 cells, 24 hours after treatment with TubastatinA at the indicated concentrations or DMSO. (**e**) Schematic representation of the experiment. (**f**) Representative images of ONS-76 cells treated as highlighted in (*e*) with the indicated siRNAs, immunostained for α-tubulin (α-TUB) and counterstained with DAPI. Scale bars: 10 μm. (**g**) ONS-76 cells were treated as described in (*f*), using the indicated siRNAs, and the percentage of binucleated cells was determined. Each dot indicates an independent biological replicate. All immunofluorescence quantifications were based on at least four independent biological replicates; >300 cells were analyzed per condition in each replicate. Error bars, SEM. **P<0.01, ***P<0.001; unpaired two-tailed Student's t-test. A.U., arbitrary units

SUPPLEMENTARY FIGURE 3 | Tubastatin A reduces nuclear HDAC6 and recovers BRCA1 foci and DNA damage in CITK-depleted cells.

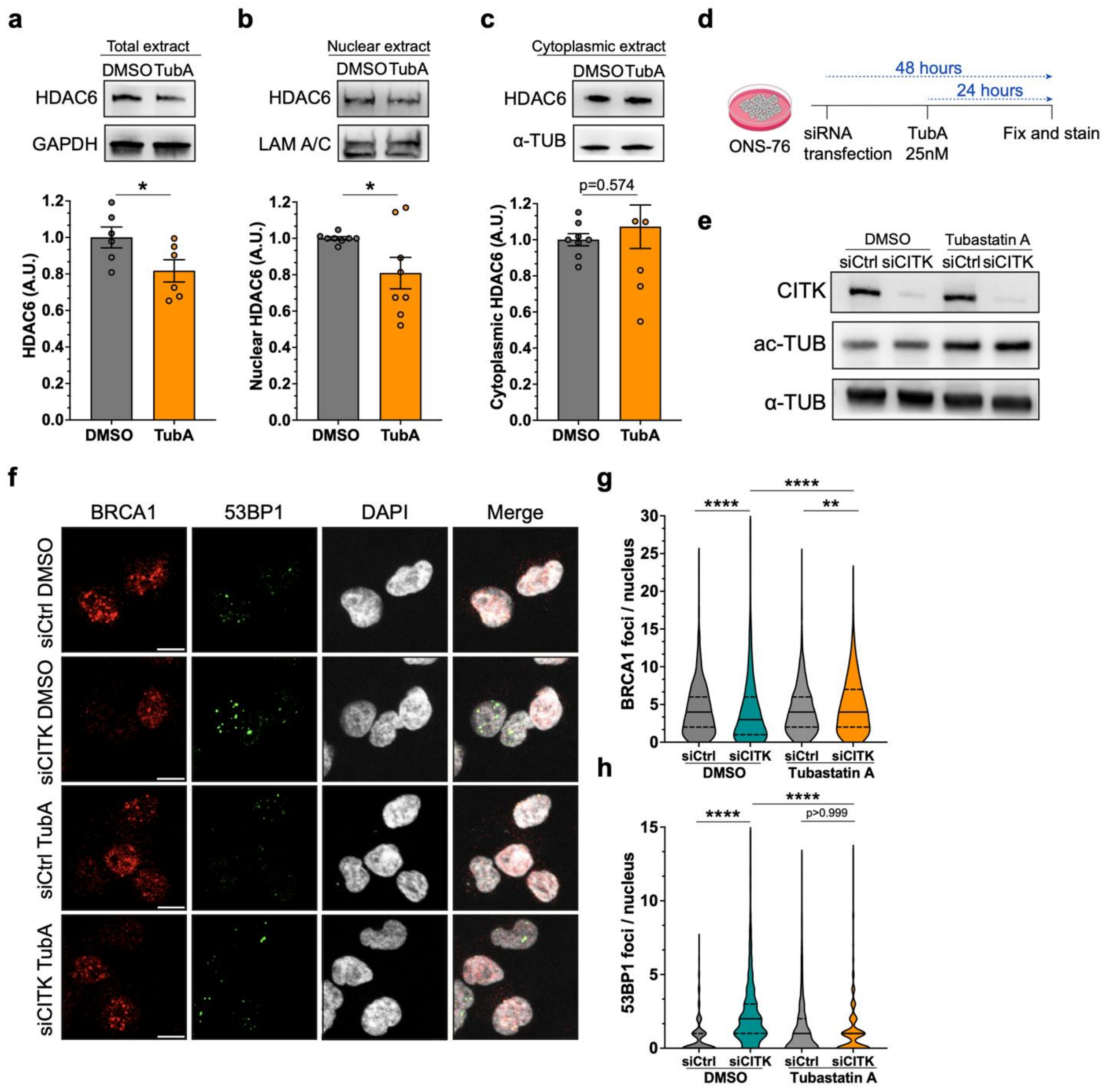

(**a**) Western blot analysis of total lysate from ONS-76 24 hours after treatment with 25nM Tubastatin A or vehicle (DMSO). The levels of HDAC6 were analyzed, and the internal loading control was GAPDH. Quantification of HDAC6 levels relative to DMSO. (**b-c**) Nuclear (*b)* and cytoplasmic (***c***) fractionation of ONS-76 24 hours after treatment with 25nM Tubastatin A or DMSO. The levels of HDAC6 were analyzed, and the internal loading controls were lamin A/C (LAM A/C) for the nucleus and α-tubulin (α-TUB) for the cytoplasm. Lower panels show the quantification of HDAC6 relative densities in the nucleus and the cytoplasm. (**d**) Schematic representation of the experiment. (**e**)

Western blot analysis of total lysate from ONS-76 cells treated as shown in (*d*), using the indicated siRNAs. The levels of CITK and acetylated tubulin (ac-TUB) were analyzed and the internal loading control was α-tubulin (α-TUB). (**f**) Representative images of ONS-76 cells treated as in (*e*), immunostained for BRCA1 and 53BP1 and counterstained with DAPI. Scale bars: 10 μm. (**g-h**) Quantification of BRCA1 (*g*) and 53BP1 (*h*) foci per nucleus in the indicated conditions. Each dot indicates an independent biological replicate. All immunofluorescence quantifications were based on at least four independent biological replicates; >300 cells were analyzed per condition in each replicate. Error bars, SEM. *P<0.05, **P<0.01, ***P<0.001; ****P<0.0001 unpaired two-tailed Student's t-test for western blots; one-way ANOVA test followed by Tukey's correction for foci. A.U., arbitrary unit.

SUPPLEMENTARY FIGURE 4 | Paclitaxel recovers BRCA1 levels and reduces DNA damage in CIT K depleted cells

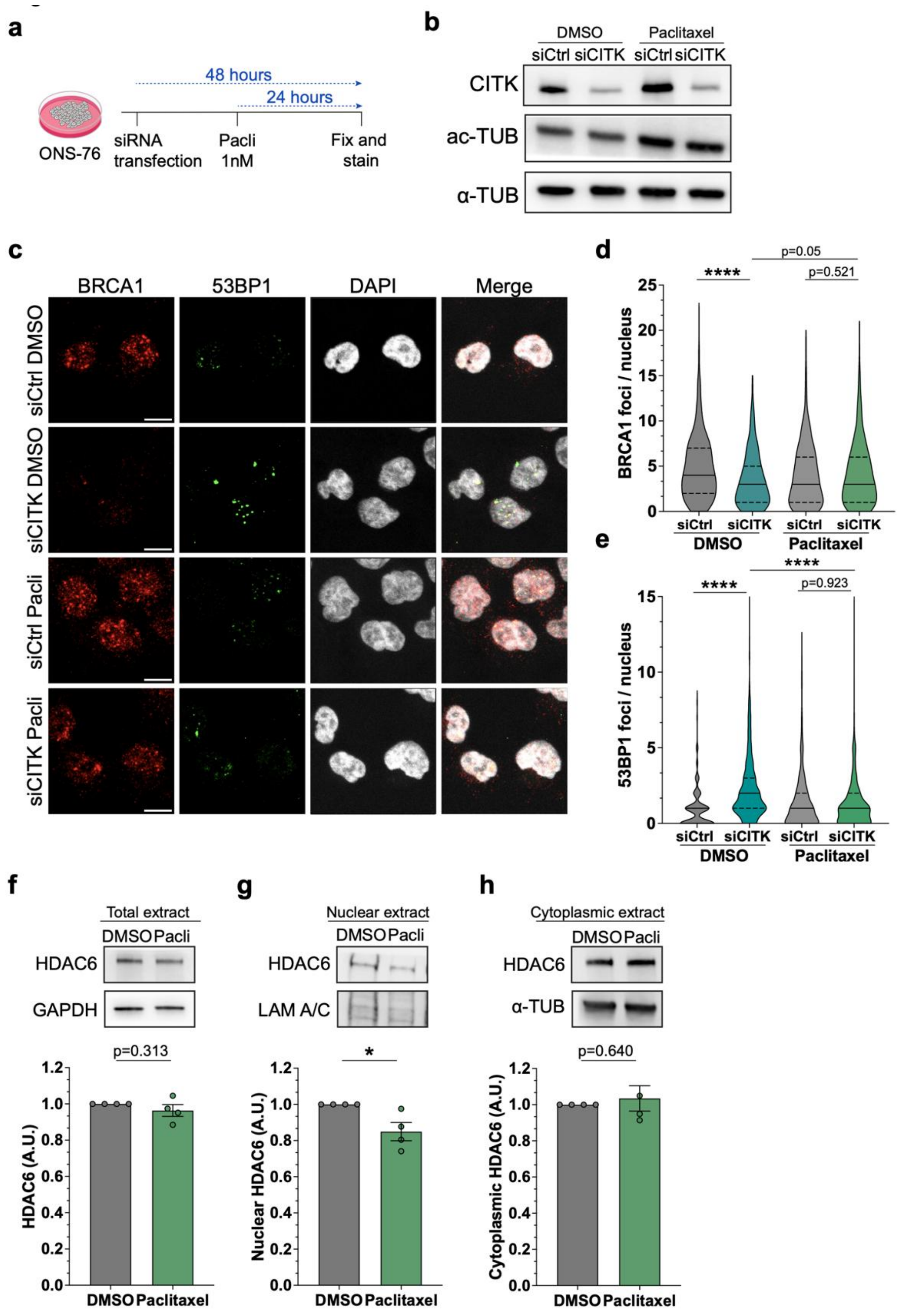

(**a**) Schematic representation of the experiment. (**b**) Western blot analysis of total lysates obtained from ONS-76 cells transfected with the indicated siRNAs and treated as described in (*a*). The levels of CITK and acetylated tubulin (ac-TUB) were analyzed and the internal loading control was α-tubulin

(α-TUB). (**c**) Representative images of ONS-76 cells treated as in (*b*), immunostained for BRCA1 and 53BP1 and counterstained with DAPI. Scale bars: 10 μm. (**d-e**) Quantification of BRCA1 (*d*) and 53BP1 (*e*) foci per nucleus in experiments performed as for panel (*c*). (**f**) Western blot analysis of total lysate obtained from ONS-76 cells, 24 hours after treatment with 1nM Paclitaxel or vehicle control (DMSO). The levels of HDAC6 were analyzed and the internal loading control was GAPDH. Quantification of HDAC6 levels relative to DMSO. (**g-h**) Nuclear (*g*) and cytoplasmic (*h*) fractions of cells treated and analyzed as described in (*f*). All immunofluorescence quantifications were based on at least four independent biological replicates; >300 cells were analyzed per condition in each replicate. Each dot in western blots quantifications indicates an independent biological replicate. Error bars, SEM. *P<0.05, ****P<0.0001; unpaired two-tailed Student's t-test for western blots; one-way ANOVA test followed by Turkey's correction for foci. A.U., arbitrary unit.

SUPPLEMENTARY FIGURE 5 | Strategies for *cita* knock-down and transient knockout in zebrafish embryos.

a

Sequence1	vs	Sequence2	cDNA sequence identity	Protein identity	Protein similarity
zf cita		*zf citb*	52.9%	53.2%	59.7%
zf cita		mouse *Cit*	59.9%	63.3%	75.6%
zf cita		human *CIT*	55.5%	63.6%	75.9%
zf citb		mouse *Cit*	56.3%	53.7%	64.4%
zf citb		human *CIT*	38.5%	53.7%	64.5%

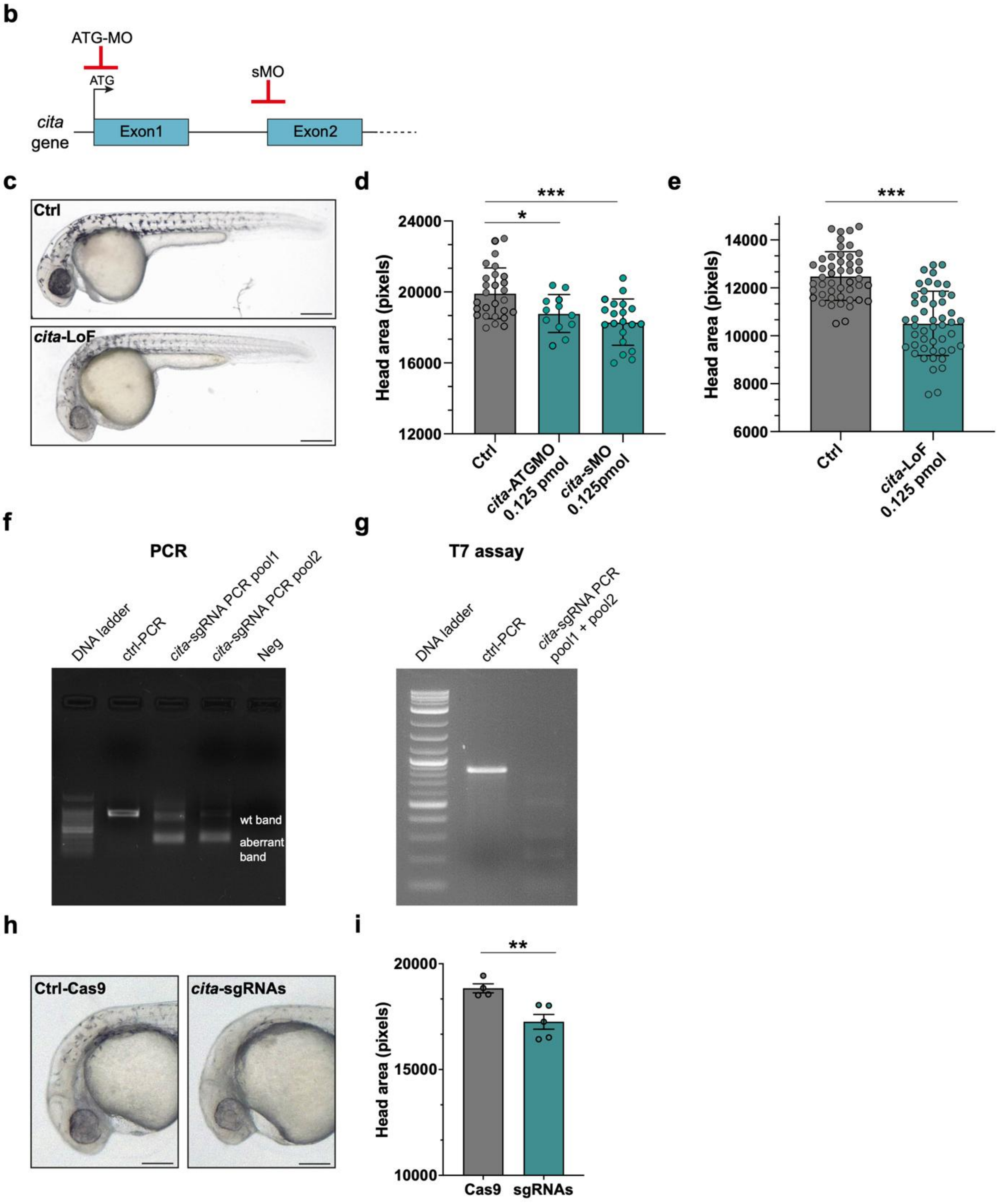

a) Results of pairwise alignments for cDNA and protein sequences (Needle-EMBOSS tool - Clustal, EMBL-EBI); cDNA sequence identity, protein sequence identity and similarity are listed in the table. (**b**) Scheme of *cita*-morpholinos targeting ATG region (*cita*-ATG-MO) and intron1-exon2 boundary (*cita*-sMO). (**c**) Representative brightfield images of 24 hpf control and *cita*-knocked down embryos. Scale bar: 100µm (**d-e**) Measurement of the head size in controls and embryos injected with *cita*-ATG-MO and *cita*-sMO in single (**d**) or in combination (**e**). (**f**) *cita* PCR amplifications of gDNA obtained from ctrl (Cas9 injected) and *cita*-sgRNAs injected embryos. To note, the intensity of the wild-type amplicon diminished *cita*-sgRNAs and a shorter band resulting from the combined action of the sgRNAs is present only in two different pools of *cita*-sgRNAs injected embryos. Neg: negative. (**g**) Results of T7-assays performed on the WT band of ctrl and *cita*-sgRNAs injected embryos. (**h**) Representative images of the head region of 24 hpf ctrl (Cas9 injected) and *cita*-sgRNAs injected embryos. Scale bar = 100µm. (**i**) Quantification of the head size (lateral area in pixel) ctrl (Cas9 injected) and *cita*-sgRNAs injected embryos. Each dot indicates an individual embryo. Error bars, SEM. *P<0.05,**P<0.01,***P<0.001 one-way ANOVA test followed by Tukey's correction or student T-test (d, e) and unpaired two-tailed Student's t-test (i).
