# Supplementary material for: CITK modulates BRCA1 recruitment at DNA double strand breaks sites through HDAC6
Source: Cell Death Dis. 2025 Apr 20;16(1):320. doi: 10.1038/s41419-025-07655-4 (PMC12009987; doi:10.1038/s41419-025-07655-4)

All blots for Figure 1h

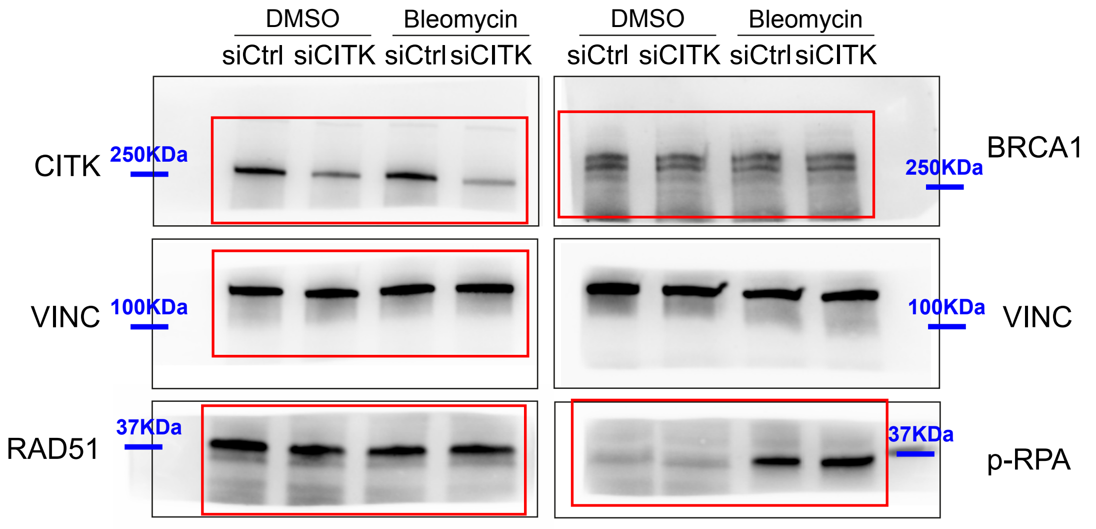

All blots for Figure 1p

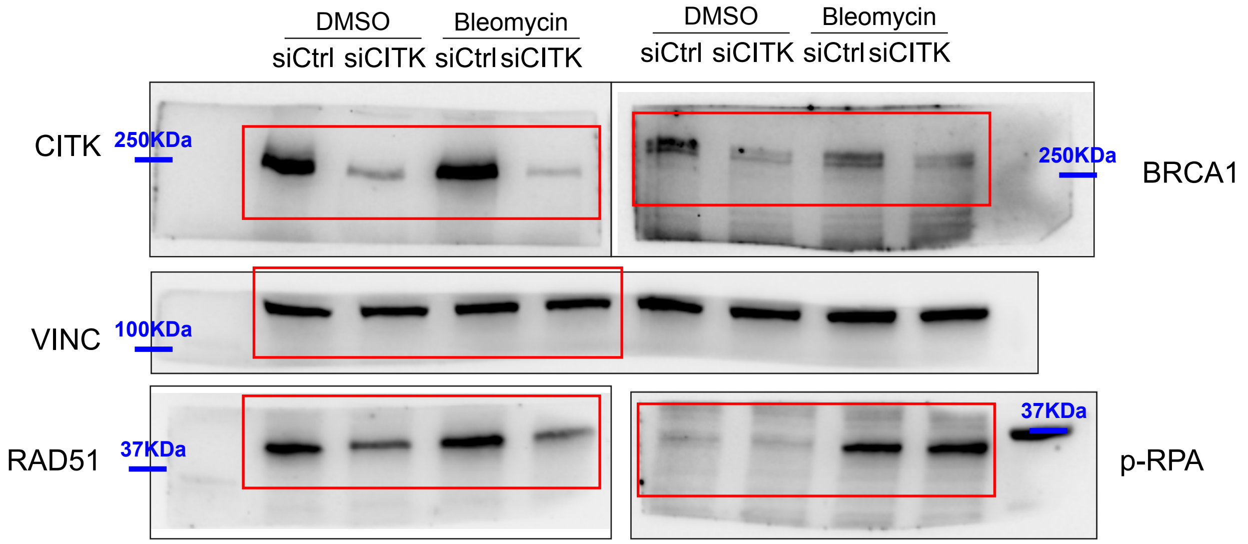

All blots for Figure 3f

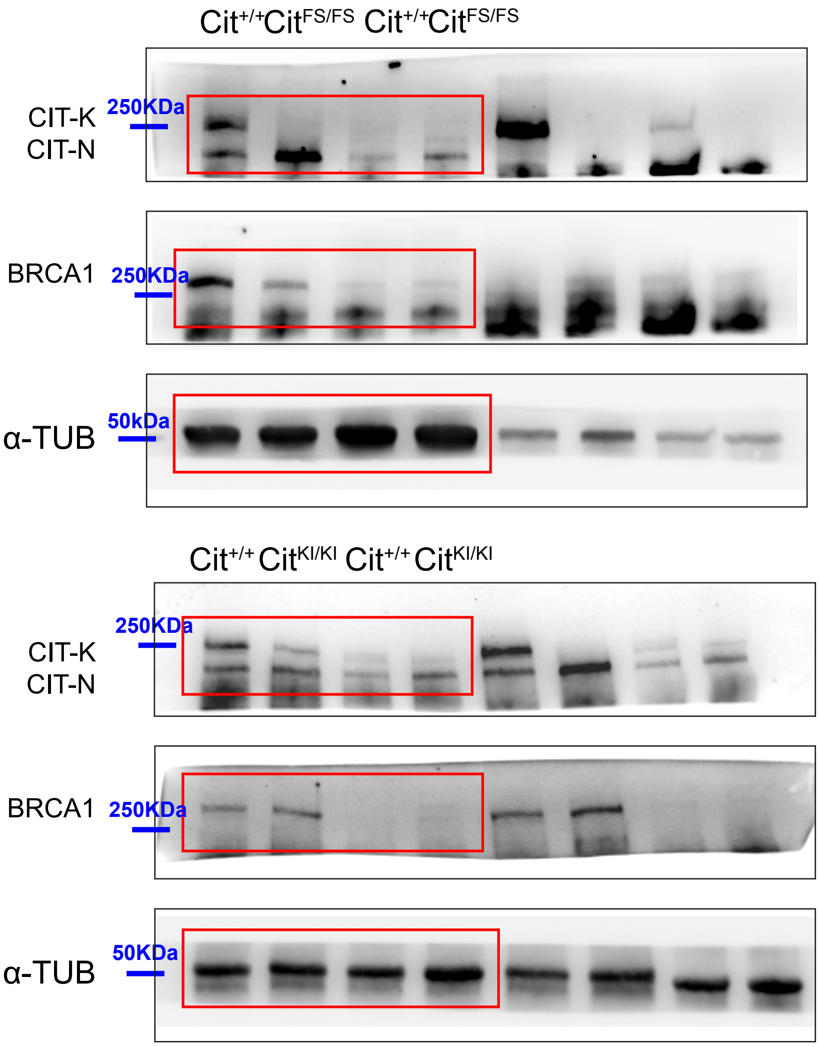

All blots for Figure 4a

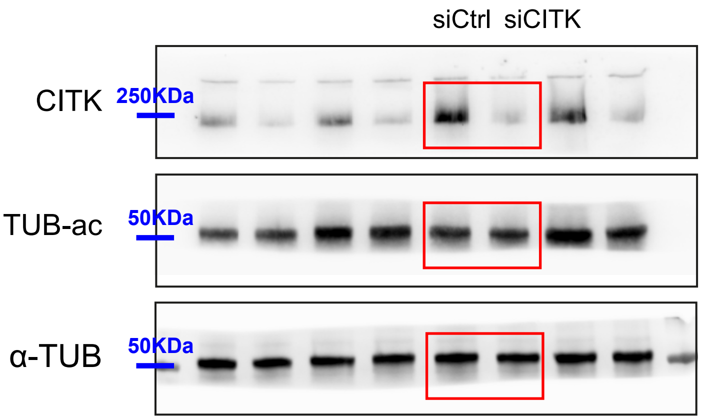

All blots for Figure 4d

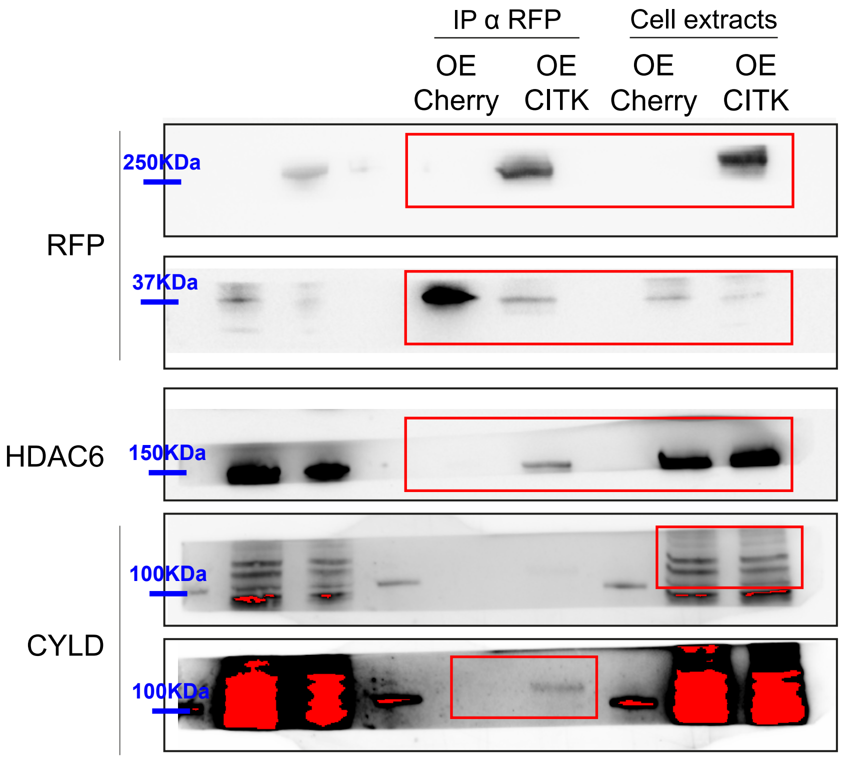

All blots for Figure 4e

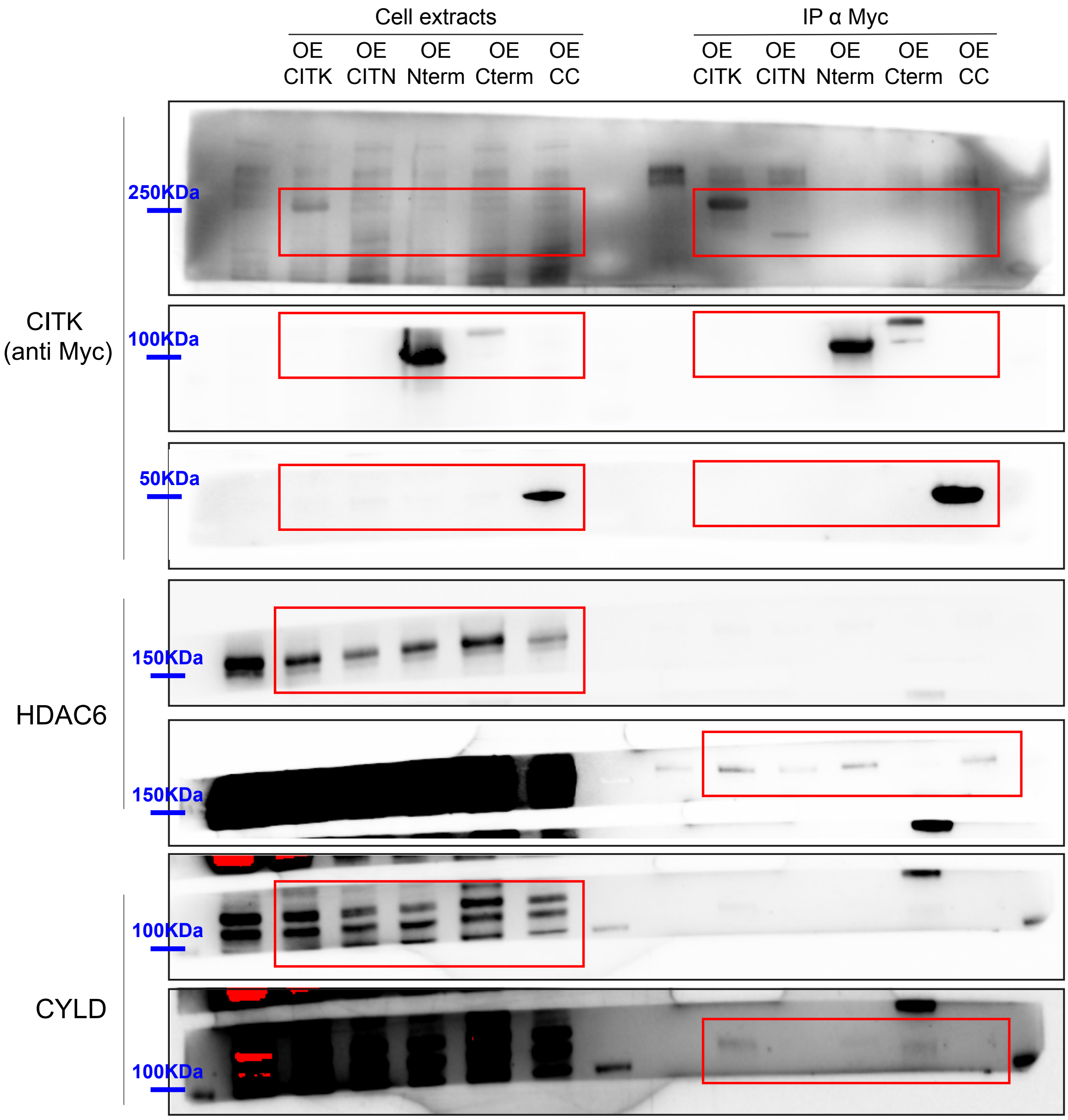

All blots for Figure 4f

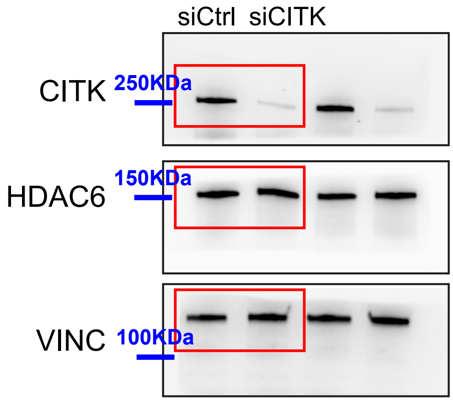

All blots for Figure 4g

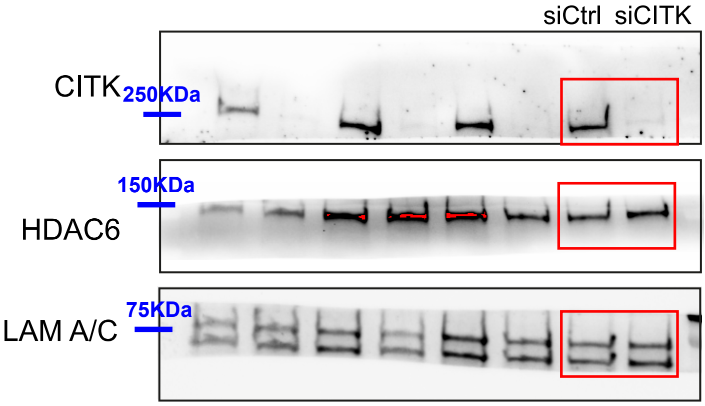

All blots for Figure 4h

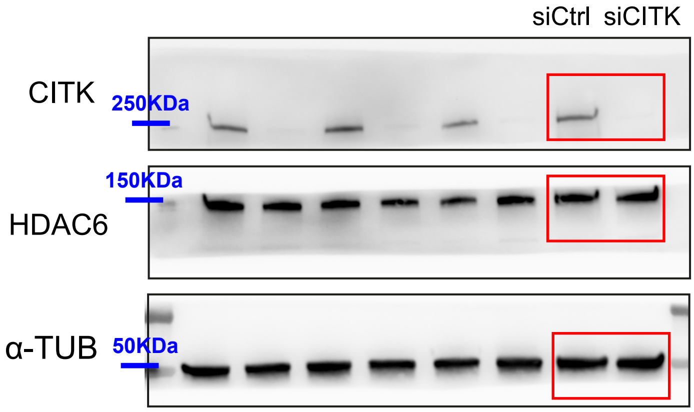

All blots for Figure 5a

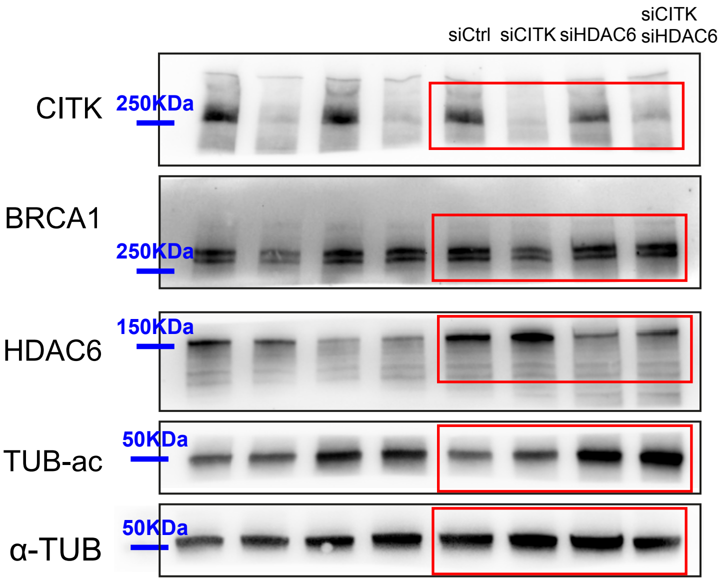

All blots for Figure S2a

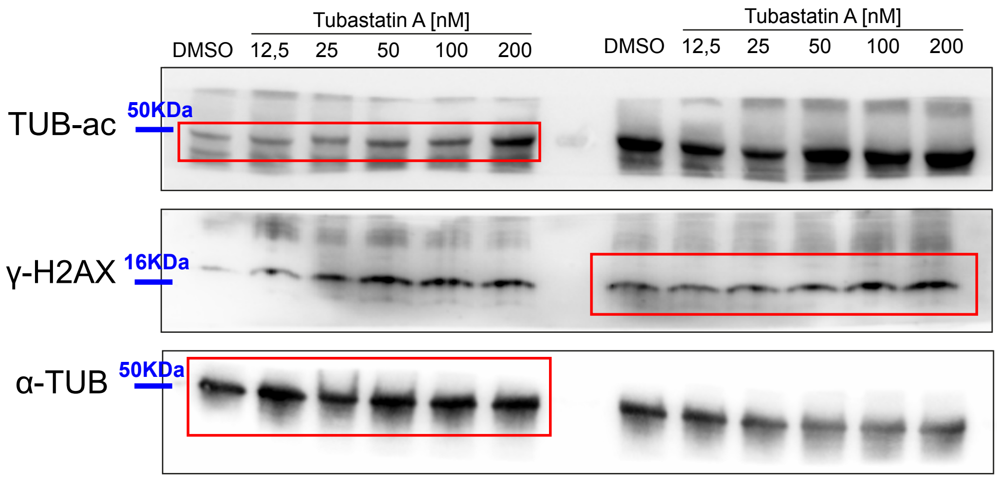

All blots for Figure S3a

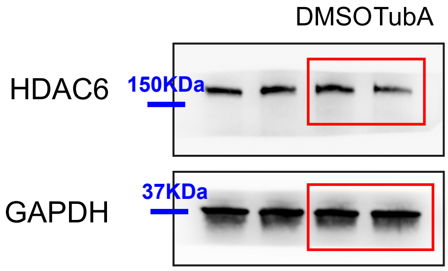

All blots for Figure S3b-c

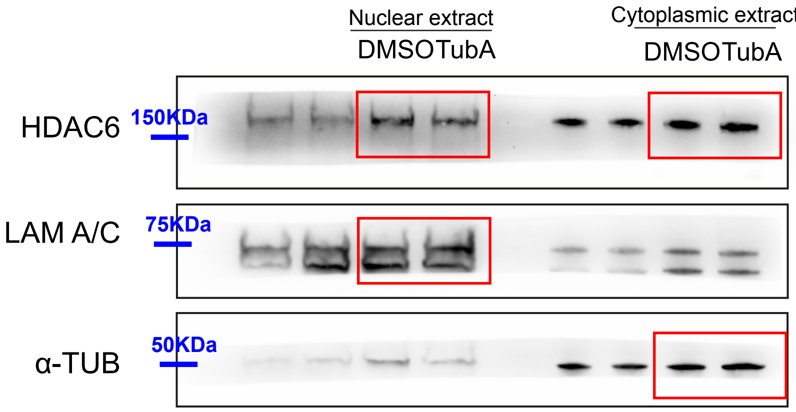

All blots for Figure S3e

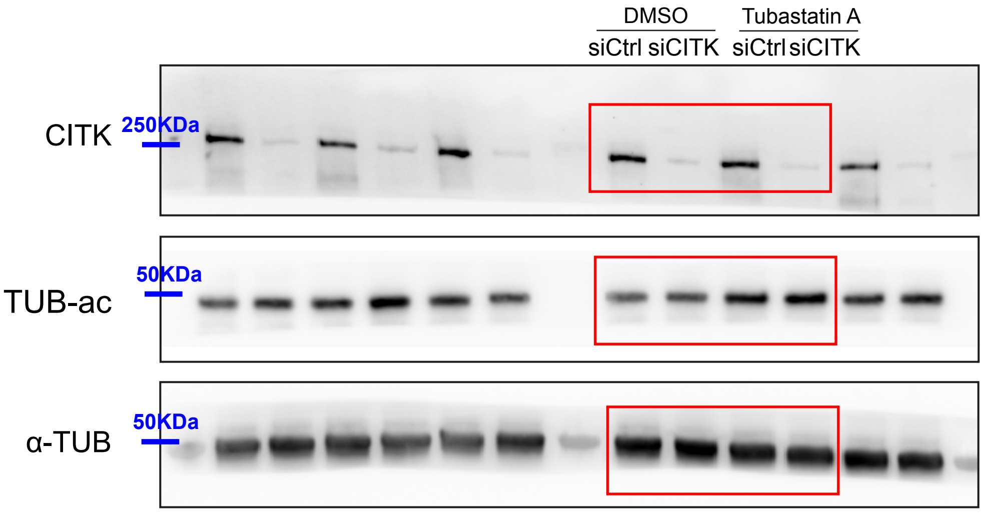

All blots for Figure S4b

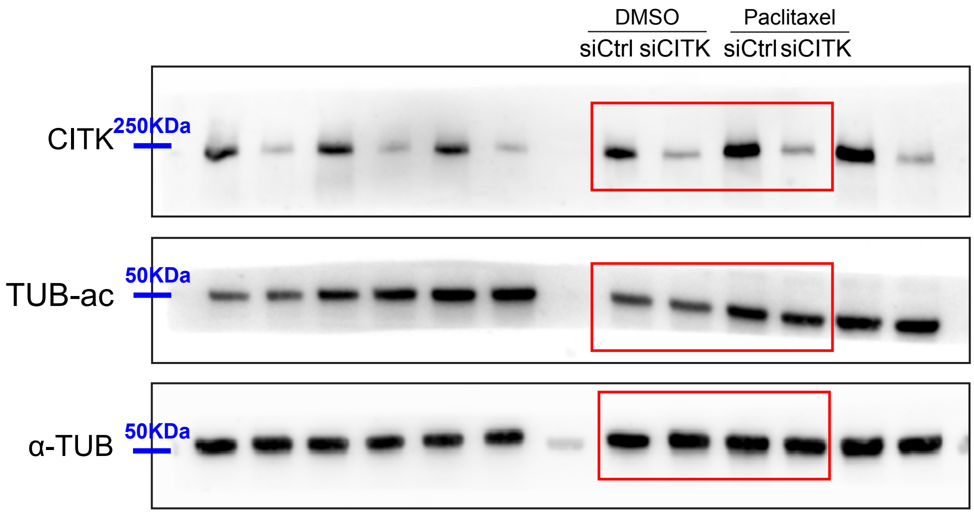

All blots for Figure S4f

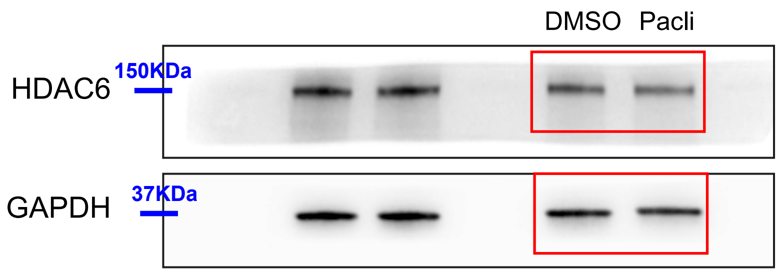

All blots for Figure S4g-h

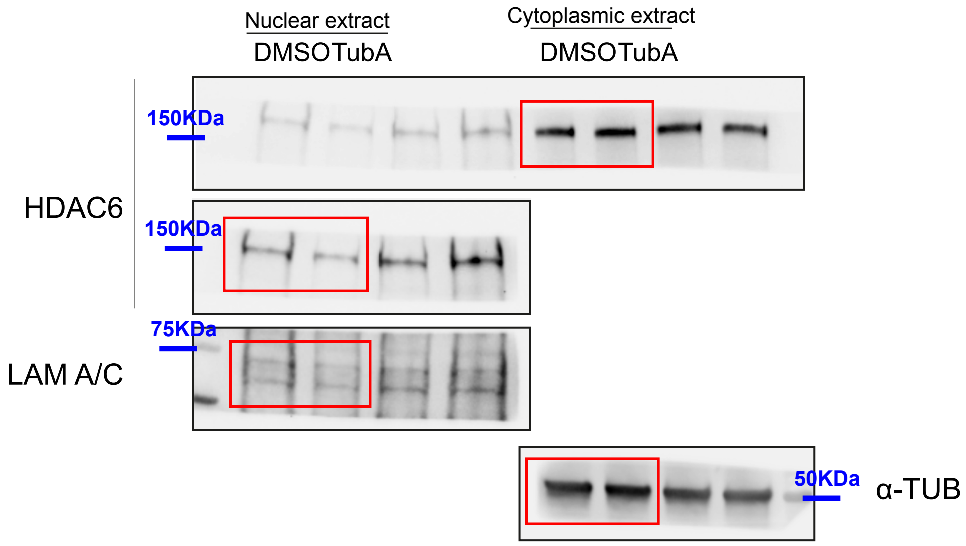

Supplement: Supplementary file 2 — Unedited gels [file 41419_2025_7655_MOESM2_ESM.pdf]
